# Phylogenetic analysis of nine *Impatiens* species from subgenus *Clavicarpa* and subgenus *Impatiens* (Sect. *Impatiens* and Sect. *Racemosae*) based on chloroplast genomes

**DOI:** 10.3389/fpls.2025.1541320

**Published:** 2025-03-27

**Authors:** Mengqing Yang, Wenxiang Lan, Jianhui Zhong, Hui Ma, Xi Huang, Meijuan Huang, Haiquan Huang

**Affiliations:** College of Landscape Architecture and Horticulture Sciences, Southwest Research Center for Engineering Technology of Landscape Architecture (State Forestry and Grassland Administration), Yunnan Engineering Research Center for Functional Flower Resources and Industrialization, Research and Development Center of Landscape Plants and Horticulture Flowers, Southwest Forestry University, Kunming, Yunnan, China

**Keywords:** *Impatiens*, chloroplast genome, comparative analysis, phylogenetic relationships, taxonomic study

## Abstract

**Introduction:**

The genus *Impatiens L.* ( Balsaminaceae) is one of the three most important bedding plant genera globally, valued for its medicinal, ornamental, and economic properties. However, the morphological overlap among species and the lack of genomic data have limited our understanding of their molecular phylogenetic relationships.

**Methods:**

This study involved the sequencing of the chloroplast genomes of 9 Impatiens species, including *Impatiens lateristachys, Impatiens siculifer* var. *porphyrea, Impatiens apalophylla, Impatiens pritzelii, Impatiens menghuochengensis, Impatiens membranifolia, Impatiens qingchengshanica, Impatiens aquatilis*, and *Impatiens racemosa*. The study evaluated sequence divergence by comparing genomic features, repeat sequences, codon usage, IR expansion and contraction, sequence alignment, and selective pressures. It then constructed phylogenetic relationships using the maximum likelihood method, revealing the evolutionary relationships among these species.

**Results:**

The results indicated that the chloroplast genome sizes ranged from 151, 784 bp (*I. racemosa*) to 152, 628 bp (*I. apalophylla*), encoding between 108-115genes[77 to 81 protein-coding genes, 27 to 30 tRNA genes, and 4 rRNA genes]. Additionally, A detailed analysis was performed on the characteristics of repeat sequences, codon preferences, and IR region. Coding sections were more conserved than non-coding regions, and the IR regions were more conserved than the LSC and SSC regions, according to sequence variation and mutation hotspot analyses. The 9 species of *Impatiens* were classified into subgenus *Clavicarpa* and subgenus *Impatiens*, including the sections *Impatiens* and Racemosae, according to the phylogenetic tree.

**Discussion:**

This study presents the chloroplast genomes of 9 species within the genus *Impatiens*, marking a novel attempt at using phylogenetic analysis to determine the taxonomic positions of *Impatiens* species. It provides new molecular evidence for the systematic and evolutionary studies of *Impatiens* species.

## 1 Introduction

The genus *Impatiens* L. was established by the botanist Carl Linnaeus in 1753 (Linnaeus, 1753). It belongs to the order Ericales, which includes only two genera: *Hydrocera* and *Impatiens*. *Impatiens* plants are annual or perennial herbaceous species within the genus *Impatiens* of the Balsaminaceae. They hold significant ornamental, economic, and medicinal value (Yu, 2008; Gao, 2012). The genus *Impatiens* has a broad distribution, primarily across tropical and subtropical regions, extending into tropical Africa, Southwest Asia, southern China, Europe, Russia, and temperate areas of North America (Grey-Wilson, 1989; Yu, 2012). *Impatiens* species exhibit diverse morphology and flower colors and are recognized as among the top three ornamental plants for flowerbeds and borders. Notably, *I. hawkeri* and *I. walleriana* are frequently utilized in horticulture, while many other species remain in the wild or are yet to be fully developed (Yu, 2012). China, particularly the karst regions, is recognized as the origin and center of diversification for the Balsaminaceae family. In Guizhou, Yunnan, and Guangxi, there are about 250 wild *Impatiens* species known to exist, many of these are utilized as supplements or medicines. In ancient China, *Impatiens* was known as “zhijiahua” and was ground into a paste for coloring nails (Cai et al., 2013). It was also referred to as “tougucao” and “jixingzi,” annual herbs used to cure onychomycosis, paronychia, rheumatism, beriberi, bruising, discomfort, and warts (Jiang et al., 2017; Kim C. S. et al., 2017).

The stems of *Impatiens* species are typically fleshy and succulent, characterized by thick leaves and delicate, membranous, fragile flowers. Floral parts often overlap and adhere during specimen pressing, making their separation and reconstruction difficult (Grey-Wilson, 1989). At the macroscopic level, the flowers, capsules, and seeds of *Impatiens* exhibit significant diversity. Consequently, the shape and size of the sepals, petals, capsules, and seeds are critical for species identification within this genus (Chen, 2001). In contrast to macroscopic features, the microstructural characteristics of pollen and seeds are less affected by environmental conditions (Janssens et al., 2012). Currently, the analysis of microstructural traits, such as pollen, leaf epidermis, and seed coat, has served as an important reference for taxonomy and phylogeny discussions (Guo, 2017; Shui et al., 2011; Pechimuthu et al., 2024). Molecular phylogenetics has significantly advanced our understanding of relationships within *Impatiens*. Fujihashi et al. (2002) published the first molecular phylogeny of *Impatiens*. This study, based on *ITS* (Internal Transcribed Spacer) sequences from 111 species, provided significant phylogenetic insights through a second independent molecular analysis (Yuan et al., 2004). Existing molecular classification studies have primarily focused on several chloroplast regions, including coding genes such as *matK*, *rbcL*, and *trnK*, as well as intergenic spacers like *atpB-rbcL* and *trnL-trnF* (Shajitha et al., 2016; Janssens et al., 2006). These studies have limited datasets and focus on a small number of samples with distinct regional characteristics.

For example, Rahelivololona et al. (2018) performed molecular phylogenetic analysis of 33 *Impatiens* species from Madagascar using nuclear and plastid data. However, molecular phylogenetic studies alone cloud not determine the distribution of morphological traits across evolutionary branches, nor can they fully capture the species diversity and complex phylogenetic relationships within *Impatiens*. In 2012, Yu et al. classified *Impatiens* into eight groups based on morphological traits and published the book “Balsaminaceae of China” (Yu, 2012). In 2016, Yu et al. proposed a new classification of *Impatiens* using sequencing data from three major genetic regions—nuclear *ITS*, chloroplast *atpB-rbcL*, and *trnL-F*—dividing the genus into two subgenera and seven groups. This classification provided significant data support for *Impatiens* resource taxonomy (Yu et al., 2016). To date, all molecular data are still derived from short sequences, many of which originate from samples with distinct regional characteristics. This limits the inferences for classification and phylogeny. For species exhibiting morphological diversity and controversial classification, molecular data alone are insufficient to provide conclusive evidence. Therefore, comprehensive studies integrating both morphological and molecular data are urgently needed to support the taxonomy and phylogeny of *Impatiens*. This study aims to resolve the phylogenetic relationships of *Impatiens* at higher resolution by using complete chloroplast genome sequences, thereby advancing taxonomy and updating research on the genus.

Chloroplasts are commonly found in the cytoplasm of higher plants and are responsible for synthesizing proteins, fatty acids, starch, pigments, and other compounds. They contain an independent and complete semi-autonomous genetic system known as the chloroplast genome (Janssens et al., 2016). Due to their ability to self-replicate, maternal inheritance, and relative conservatism, chloroplast genomes have become a significant tool in systematic research (Li et al., 2018). The chloroplast genomes of most angiosperms consist of a pair of inverted repeat sequences (IR), a large single-copy region (LSC), and a small single-copy region (SSC), with lengths ranging from 115 kb to 165 kb (Wang et al., 2024; Sun et al., 2024; Huang et al., 2024a). These genomes typically contain 110 to 113 genes, which are involved in gene expression (such as tRNA and rRNA genes), photosynthesis, and other metabolic functions (Zhou et al., 2020). The highly conserved nature of chloroplast genomes provides valuable molecular evidence for phylogenetic analysis and plant classification. Moreover, chloroplast genomes are useful for molecular markers, genetic modification, and plant barcode recognition (Gu et al., 2018). A detailed comparison of the chloroplast genomes of *Impatiens* species can deepen our understanding of their phylogenetic relationships and provide important insights for classification and evolutionary studies. Despite previous studies that utilized certain chloroplast genes (such as *matK*, *rbcL*, *trnK*, *trnL-trnF*, and *atpB-rbcL*) to investigate the phylogeny of *Impatiens* species, challenges remain (Shajitha et al., 2016; Yu et al., 2016). Current research primarily focuses on species that are unique to specific regions, leading to classification controversies for some morphologically complex species (such as *I. lateristachys*) due to unresolved phylogenetic relationships. The existing molecular data are based solely on a limited number of short gene sequences, which constrains their ability to resolve complex phylogenetic relationships.

Using entire chloroplast genomes, this study presented the chloroplast genomes of 9 species in the genus *Impatiens*, marking a novel attempt at phylogenetic analysis to determine the taxonomic position of *Impatiens* species. The objectives of this study were: (i) to conduct a comprehensive analysis of the chloroplast genomes of *Impatiens* species, including basic structural information of the chloroplast genomes, characteristics of repetitive sequences, codon usage preferences, region of inverted repeats (IR) expanding and contracting, comparative genomic differences, mutation hotspots, and analysis of selective pressures among species. (ii) to further understand the phylogenetic relationships of *Impatiens* species. (iii) to construct a phylogenetic tree based on complete chloroplast genomes and conduct taxonomic analysis. Research on the phylogeny, taxonomy, population genetics, and genetic engineering of *Impatiens* species can use this paper as a reference. It offers important information for the systematics and evolutionary studies of *Impatiens* species.

## 2 Materials and methods

### 2.1 Materials and DNA extraction

9 *Impatiens* species samples were gathered from different sites (**Supplementary Table S1**) and kept at Southwest Forestry University’s Plant Laboratory in Kunming, Yunnan Province. Fresh leaves from Province were gathered, put right away in liquid nitrogen, and then kept at -80℃ until analysis. Genomic DNA was extracted using the Omega DNA Extraction Kit (Beijing, China). A spectrophotometer was used to measure the concentration of roughly 5-10 μg of genomic DNA, and electrophoresis on a 1.5% agarose gel was used to confirm the integrity of the DNA (Doyle and Doyle, 1990).

### 2.2 Library construction and sequencing

Pairwise sequencing of the chloroplast genomes of 9 *Impatiens* species was carried out utilizing the Illumina NovaSeq 6000 platform. Adapter sequences and paired-end reads with a N content greater than 10% of the total read length were eliminated from the raw data during processing. Clean sequencing data was obtained by excluding paired-end reads that had low-quality bases (Q < 5) that accounted for more than 50% of the read length.

### 2.3 Genome assembly and annotation

The chloroplast genomes of 9 *Impatiens* species were assembled using GetOrganelle v1.7.7.0 (Jin et al., 2020) with default parameters, resulting in complete circular chloroplast genome sequences. The assembled FASTA files were submitted to the online annotation tool Cpgavas2 (https://www.herbalgenomics.org/cpgavas2) (Shi et al., 2019) to obtain relevant sequence information for the chloroplast genomes.

### 2.4 Basic information analysis and physical map construction

The web program Bioinformatics Cloud (http://cloud.genepioneer.com:9929) was used to determine the GC content of four sections of the chloroplast genomes of 9 *Impatiens* species (Tong et al., 2022). To create chloroplast genome maps, the annotated GBF data were put into the web program Chloroplot (https://irscope.shinyapps.io/Chloroplot/) (Zheng et al., 2020).

### 2.5 SSR sequence analysis and analysis of scattered repeats

The chloroplast genomes of 9 *Impatiens* species were subjected to simple sequence repeat (SSR) analysis using the web application MISA (https://webblast.ipk-gatersleben.de/misa/index.php) (Thiel et al., 2003). The parameters for repeat units of one to six nucleotides were set to 10, 6, 4, 3, 3, and 3, with a minimum distance of 100 bp between two SSRs. The online software REPuter (https://bibiserv.cebitec.uni-bielefeld.de/reputer) (Kurtz et al., 2001) was utilized to analyze the scattered repeats in the cpDNA of 9 *Impatiens* species. The parameters were set as follows: the minimum repeat size was set to 30 bp, the Hamming distance was set to 3, and the sequence identity was set to 90%.

### 2.6 Codon preference analysis

Codon usage frequencies of synonymous codons in the cpDNA of 9 *Impatiens* species were calculated using CodonW 1.4.4 (Langmead et al., 2009) with default parameters. A codon heatmap was generated and enhanced using TBtools.

### 2.7 Comparative analysis of IR regions and genome similarity analysis

The boundary regions of the large single-copy (LSC), inverted repeat (IR), and small single-copy (SSC) regions of the cpDNA of 9 *Impatiens* species were compared using the CPJSdraw boundary mapping tool available on the online platform GenePioneer (http://cloud.genepioneer.com:9929). This analysis focused on the variations in the positions of these regions. The online tool mVISTA (http://genome.lbl.gov/vista/index.shtml) (Frazer et al., 2004) was used to visualize the cpDNA of 9 *Impatiens* species, taking *Hydrocera triflora* as a reference sequence under the Shuffle-LAGAN model. This comparison focused on the differences among exons, introns, non-coding regions, and coding regions within the chloroplast genomes, thereby highlighting conserved and variable regions among species.

### 2.8 Nucleotide polymorphism analysis and selection pressure analysis

MAFFT software (Katoh and Standley, 2013) was used to align the FASTA files of the 9 species, followed by manual correction using MEGA7 (Kumar et al., 2016). Nucleotide diversity analysis was conducted using DnaSP5 software (Rozas et al., 2017). Using *Hydrocera triflora* as the reference species, the CPStools (Huang et al., 2024b) package was downloaded and installed via Python software. The Ka/Ks analysis module of CPStools was then employed to import GenBank (GB) files for analysis. Subsequently, a clustering heatmap of Ka/Ks values was generated using TBtools (Chen et al., 2023) software to visually present the results.

### 2.9 Phylogenetic analysis

The chloroplast genome sequences of 24 species from the Ericales order were selected, including the 9 *Impatiens* species newly sequenced in this study and an additional 15 species downloaded from NCBI. These 14 Ericales species comprised 11 from the *Impatiens* genus, one from the *Hydrocera* genus, and one species each from the Primulaceae and Actinidiaceae (**Supplementary Table S12**). Using the Primulaceae and Actinidiaceae as outgroups, chloroplast genome sequences were aligned using the online MAFFT tool (https://www.ebi.ac.uk/Tools/msa/mafft/). After successful alignment, Gblocks (http://www.phylogeny.fr/one_task.cgi?task_type=gblocks) was used to trim the conserved regions. Phylogenetic trees were then reconstructed using the maximum likelihood method implemented in IQtree (Nguyen et al., 2015), with default parameters (1000 iterations, 1000 bootstraps, and model selection). The best-fit model GTR+F+I+R5 was used to construct the tree, and the results were visualized and refined using Figtree software (http://tree.bio.ed.ac.uk/software/figtree).

## 3 Results

### 3.1 Structure and characteristics of the chloroplast genomes in *Impatiens*

**Figure 1:**
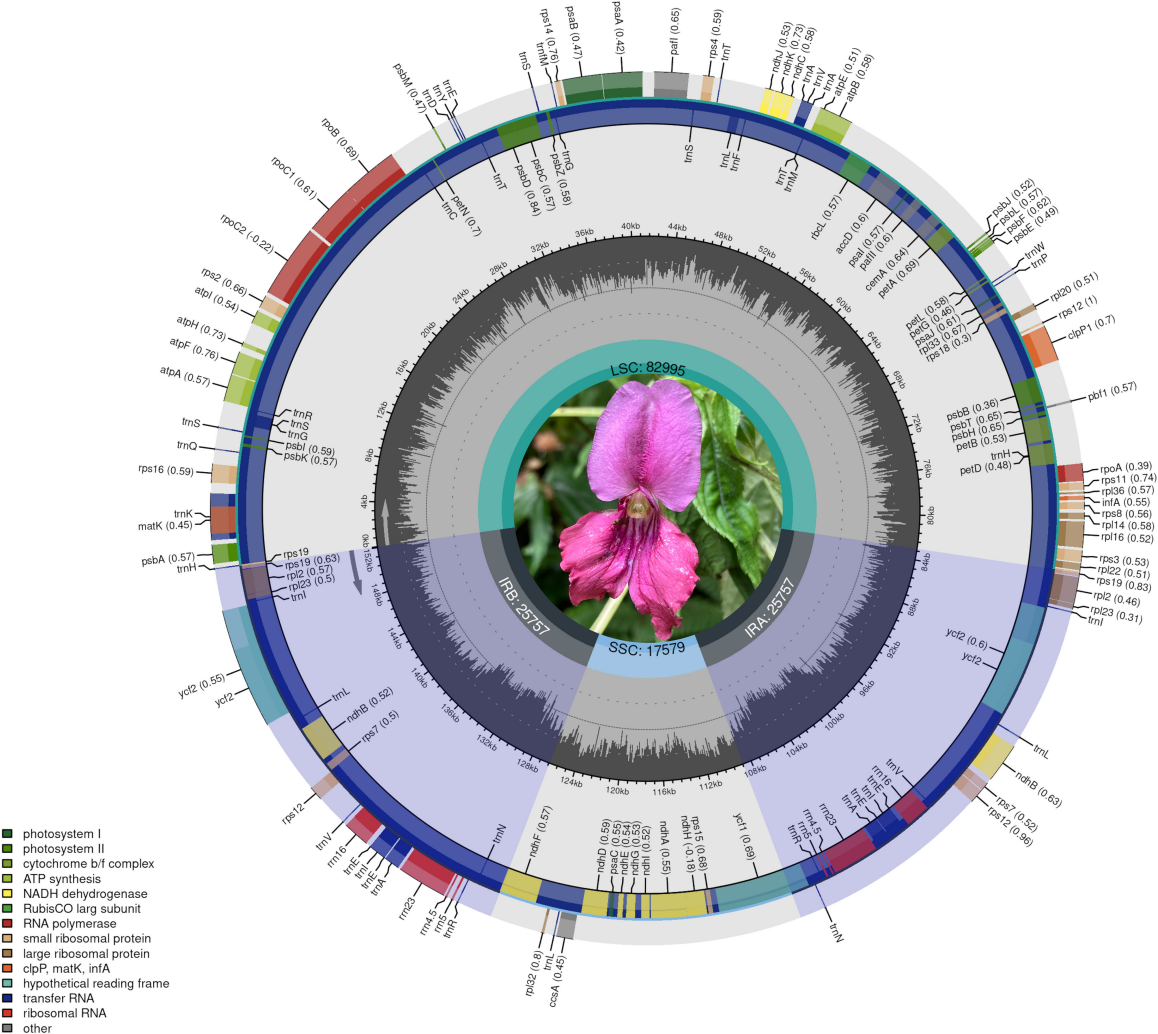
Gene map of *I. lateristachys.* The genes located outside the map are transcribed in a clockwise direction, while those inside are transcribed counterclockwise.

The chloroplast genomes of the 9 *Impatiens* species analyzed were typical circular DNA molecules, with GC content in each region consistent with previously published data on *Impatiens* chloroplast genomes. The chloroplast genomes had an average GC content of 37%, with sizes ranging from 151, 784 bp in *I. racemosa* to 152,628 bp in *I. apalophylla*. The large single-copy (LSC) region sizes ranged from 82, 995 bp in *I. lateristachys* to 83, 480 bp in *I. siculifer* var. *porphyrea*), with a GC content of 34% to 35%. The small single-copy (SSC) regions exhibited GC content of 29-30%, with sizes ranging from 17, 253 bp in *I. pritzelii* to 17, 893 bp in *I. qingchengshanica*. The inverted repeat (IR) region sizes ranged from 25, 535 bp in *I. racemosa* to 25, 883 bp in *I. aquatilis*, with a GC content of 43%. The average AT content across the chloroplast genomes of the 9 *Impatiens* species was 63.12%, while the average GC content was 36.88%. No significant interspecific difference in GC content were observed among the basic structural regions (LSC, IR, and SSC). Additionally, the lowest GC contents were 29.26% in the SSC, 34.32% in the LSC, and 43.04% in the IR regions. The LSC and IR sections had significantly higher GC content compared to the SSC region (**Table 1**, **Figure 1**; **Supplementary Figure S1**). These results indicated that the 9 *Impatiens* species differed in the lengths of their chloroplast genomes and their GC content.

**Table 1:** Characteristics of complete chloroplast genomes for 9 *Impatiens* species.

Species	Genome size(bp) and content of GC(%)		LSC size (bp) and content of GC(%)		SSC size (bp) and content of GC(%)		IR size (bp) and content of GC(%)	
*I. lateristachys*	152, 088	37	829, 95	34.50	175, 79	29.26	257, 57	43.10
*I. siculifer* var. *porphyrea*	152, 390	37	834, 80	34.44	177, 94	29.62	255, 58	43.08
*I. apalophylla*	152, 628	37	833, 61	34.84	178, 73	29.80	256, 97	43.10
*I. pritzelii*	152, 222	37	833, 65	34.44	172, 53	29.36	258, 02	43.00
*I. menghuochengensis*	152, 184	37	830, 78	34.61	174, 24	29.49	258, 41	43.04
*I. membranifolia*	152, 222	37	830, 98	34.60	174, 18	29.30	258, 53	43.05
*I. qingchengshanica*	152, 432	37	830, 93	34.90	178, 93	29.77	257, 23	43.07
*I. aquatilis*	152, 590	37	833, 96	34.39	174, 28	29.50	258, 83	42.91
*I. racemosa*	151, 784	37	834, 34	34.46	172, 80	29.75	255, 35	43.11

### 3.2 The function of the chloroplast genomes in *Impatiens*

The chloroplast genomes of the 9 *Impatiens* species were divided into four major groups according on gene function. The first group included genes involved in self-replication, such as tRNA-coding genes, rRNA-coding genes, RNA polymerase subunit-coding genes, as well as ribosomal small subunit protein-coding genes and ribosomal large subunit protein-coding genes. ATP synthase, photosystem I and II, cytochrome b/f complex, NADH dehydrogenase, and ribulose-1, 5-bisphosphate carboxylase genes were among the genes in the second group that were involved in photosynthesis. The third group consisted of biosynthesis-related genes, including mature enzyme genes, envelope protein genes, and ATP-dependent protease genes. The fourth group included genes of unknown function, primarily presumed chloroplast open reading frames (**Supplementary Table S2**–**S9**).

For instance, the chloroplast genome of *I. lateristachys* (**Table 2**) contained 113 genes in total, including 79 genes that coded for proteins, 30 genes that coded for transfer RNA (tRNA), and 4 genes that coded for ribosomal RNA (rRNA) (**Supplementary Table S10**). The first group of self-replication-related genes comprised 25 genes, including 9 ribosomal large subunit protein-coding genes, 12 ribosomal small subunit protein-coding genes, and 4 RNA polymerase subunit-coding genes. The second group involved 43 genes related to photosynthesis, including 5 genes for photosystem I, 14 genes for photosystem II, 6 genes for ATP synthase, 6 genes for the cytochrome b/f complex, 11 genes for NADH dehydrogenase, and 1 gene for ribulose-1, 5-bisphosphate carboxylase. The third group of biosynthesis-related genes comprised 6 genes, including mature enzyme genes, envelope protein genes, and ATP-dependent protease genes. The fourth group contained 5 genes of unknown function, 3 of which were presumed to be chloroplast open reading frames.

**Table 2:** Chloroplast genome gene information of *I. lateristachys*.

Classification of genes	Subclassification of genes based on functions	Gene name					Total numbers
Photosynthesis related genes	PhotosystemI	*psaA*	*psaB*	*psaC*	*psaI*	*psaJ*	5
	PhotosystemII	*psbA*	*psbB*	*psbC*	*psbD*	*psbE*	14
		*psbF*	*psbH*	*psbI*	*psbJ*	*psbk*	
		*psbL*	*psbM*	*psbT*	*psbZ*		
	Cytochrome b/f complex	*petA*	petB*	petD*	*petG*	*petL*	6
		*petN*					
	ATP synthase	*atpA*	*atpB*	*atpE*	atpF*(2)	*atpH*	6
		*atpI*					
	NADPH dehydrogenase	ndhA*(2)	ndhB*(2)	*ndhC*	*ndhD*	*ndhE*	11
		*ndhF*	*ndhG*	*ndhH*	*ndhI*	*ndhJ*	
		*ndhK*					
	Rubisco	*rbcl*					1
Self-replication	Transcription	*rpoA*	*rpoB*	rpoC1*	*rpoC2*		4
	Small subunit of ribosome	*rps11*	rps12**(2)	*rps14*	*rps15*	rps16*	12
		*rps18*	*rps19(2)*	*rps2*	*rps3*	*rps4*	
		*rps7*(2)	*rps8*				
	Large subunit of ribosome	rpl2*(4)	*rpl14*	rpl16*	*rpl20*	*rpl22*	9
		*rpl23*(2)	*rpl32*	*rpl33*	*rpl36*		
	Translational initiation factor	*infA*					1
	Ribosomal RNA	*rrn4.5*(2)	*rrn5*(2)	*rrn16*(4)	*rrn23*(4)		4
	Transfer RNA	trnA-UGC*(2)	*trnC-GCA*	*trnD-GUC*	*trnE-UUC*	*trnF-GAA*	30
		*trnG-GCC*	trnG-UCC*	*trnH-GUG*	*trnI-CAU*(2)	trnI-GAU*(4)	
		trnK-UUU*	*trnL-CAA*(2)	trnL-UAA*	*trnL-UAG*	*trnM-CAU*	
		*trnN-GUU*(4)	*trnP-UGG*	*trnQ-UUG*	*trnR-ACG*(2)	*trnR-UCU*	
		*trnS-GCU*	*trnS-GGA*	*trnS-UGA*	*trnT-GGU*	*trnT-UGU*	
		*trnV-GAC*(2)	trnV-UAC*	trnV-UAC*	*trnY-GUA*	*trnfM-CAU*	
0ther genes	Cytochrome c synthesis	*ccsA*					1
	RNA processing	*matK*					1
	Carbon metabolism	*cemA*					1
	Fatty acid synthesis	*accD*					1
	proteolysis	clpP1**					1
	Other	pafI**(2)	*pafII*	*pbf1*			3
Genes of unknown function		*ycf1*(2)	*ycf2*(4)				2
Total genes							113

Gene*:Gene with one introns; Gene**: Gene with two introns; Gene(2): Number of copies of multi-copy genes.

### 3.3 Analysis of repetitive sequences

**Figure 2:**
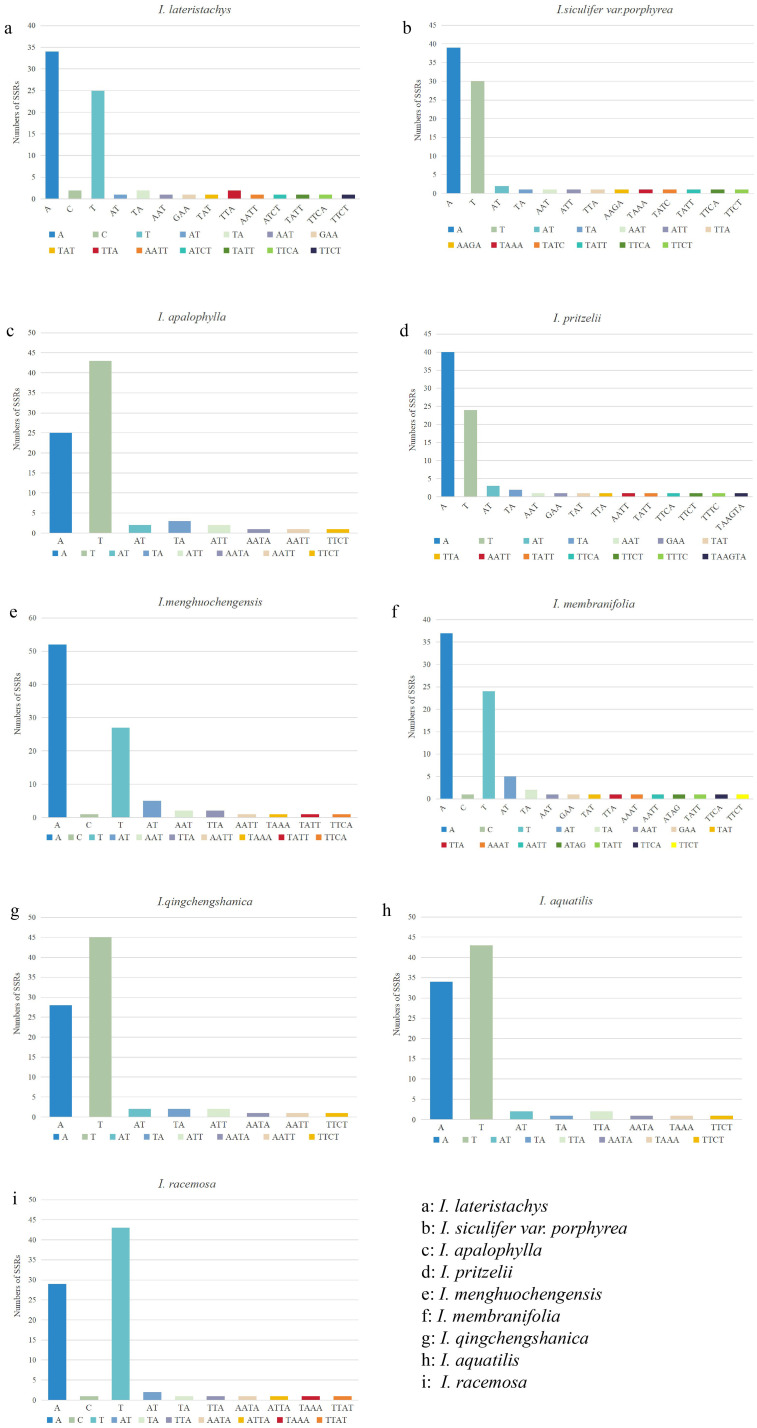
Number of SSR motifs identified in various class types. The Y-axis represents the number of SSRs, while the X-axis represents the types of SSRs. **(a)**: I. lateristachys, **(b)**: I. siculifer var. porphyrea, **(c)**: I. apalophylla, **(d)**: I. pritzelii, **(e)**: I. menghuochengensis, **(f)**: I. membranifolia, **(g)**: I. qingchengshanica, **(h)**: I. aquatilis, **(i)**: I. racemosa.

SSR analysis was performed on 9 *Impatiens* species, identifying 834 SSR sequences. Of these, 709 were mononucleotide (85.0%), 44 were dinucleotides (5.3%), 32 were trinucleotides (3.8%), 48 were tetranucleotides (5.8%), and 1 was a hexanucleotide (0.1%), with no pentanucleotides were detected. Mononucleotide sequences were the most abundant, followed by tetranucleotides, while hexanucleotides, detected only in *I. pritzelii*, were the least common. *I. menghuochengensis* had the most, while *I. lateristachys* had the fewest, at just 74 (**Supplementary Table S11**). As shown in **Figure 2**, in the 9 species of *Impatiens*, SSR repeat sequences included A/T or A/C/T as mononucleotide repeats, AT/TA as dinucleotide repeats, with AT detected solely in *I. menghuachengensis*, AAT, GAA, TAT, TTA, and TAA for trinucleotide repeats, AATT, ATCT, TATT, TTCA, TTCT, ATA, TTTC, AAAT, AATA, TAAA, ATGA, ATTA, and TTAT for tetranucleotide repeats, and the hexanucleotide repeat TAAGTA found exclusively in *I. pritzelii.* The analysis revealed that polyA and polyT comprised the majority of simple repeat sequences, with few polyG or polyC sequences. This also explained the observed A/T base preference in cpDNA, and the findings are consistent with those from simple repeat sequences in the cpDNA of other species.

**Figure 3:**
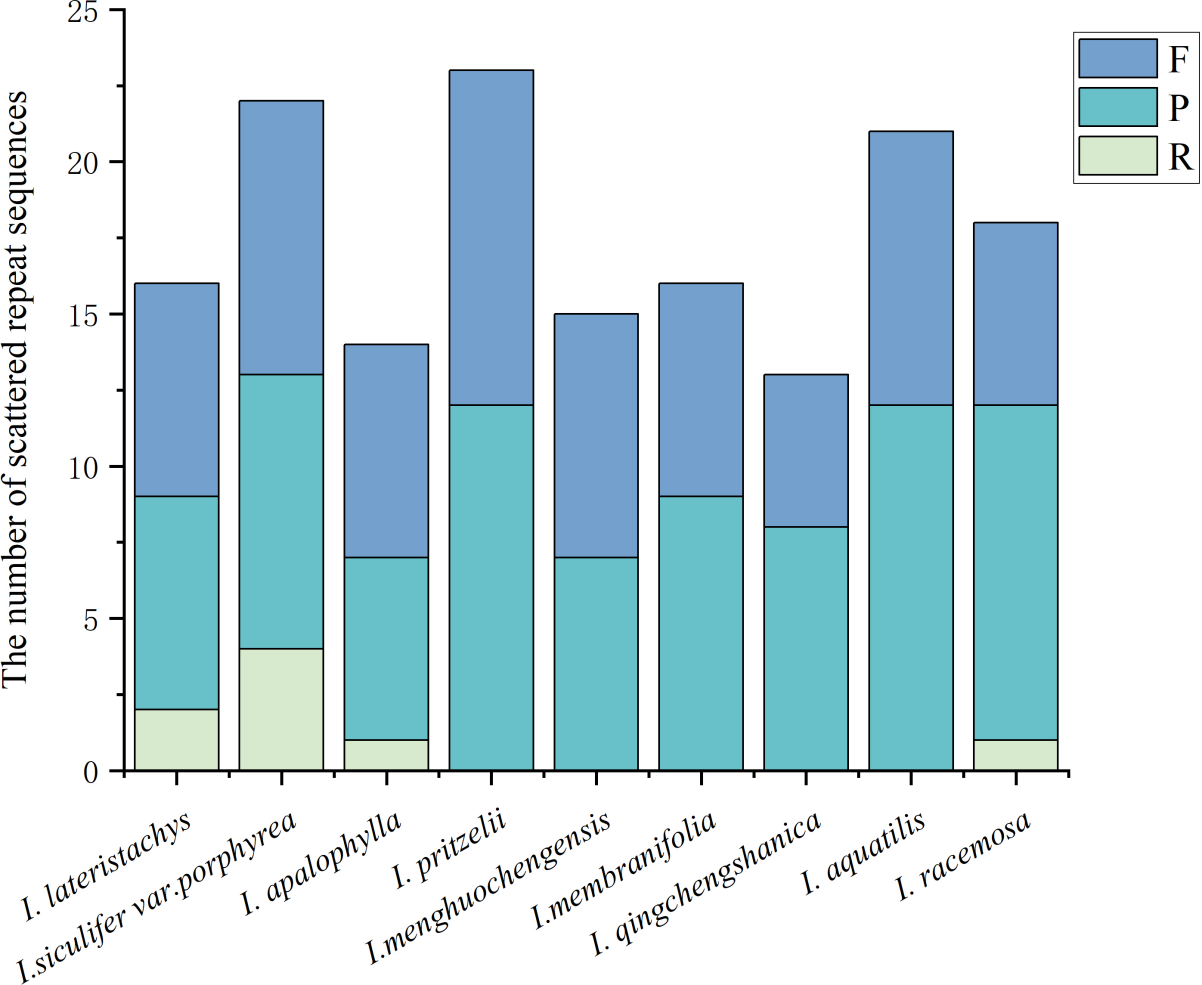
Scattered repeats of chloroplast genomes in *Impatiens* and their numbers. F: forward repeats, P: palindromic repeats, R: reverse repeats.

This study statistically examined scattered repeat sequences in the chloroplast genomes of 9 *Impatiens* species (**Figure 3**). A total of 183 pairs of repeat sequences were identified, including 10 pairs of reverse repeats (R), 89 pairs of palindromic repeats (P), and 84 pairs of forward repeats (F). *I. pritzelii* possessed 23 repeats, including 11 forward and 12 palindromic repeats, making it the species with the most repeats. In contrast, the fewest repetitions were found in *I. apalophylla*. Approximately 56.59% of the total scattered repeats were palindromic repeats, which were most abundant in *I. pritzelii* and *I. aquatilis* samples, forward repeats constituted about 37.91%. Reverse repeats were detected in *I. lateristachys*, *I. siculifer* var. *porphyrea*, and *I racemosa*. No complementary repeat sequences were identified among the 9 *Impatiens* species.

### 3.4 Analysis of codon usage bias

This study analyzed codon usage bias in the chloroplast genomes of 9 *Impatiens* species, revealing the use of 64 codons. In protein-coding genes, the number of codons ranged from 50,594 in *I. racemosa* to 50,876 in *I. apalophylla* (**Table 3**). As in most angiosperms, the most prevalent codon among these 9 *Impatiens* species encoded leucine (Leu), while the codon UUU, which encodes phenylalanine (Phe), was the most frequent, ranging from 2,296 (*I. apalophylla*) to 2,433 (*I. qingchengshanica*). In contrast, the codons GCG (for alanine) and CGC (for arginine) were the least abundant, with counts ranging from 217 (*I. racemosa*) to 230 (*I. qingchengshanica*). The termination codon UAA was the most abundant (**Table 3**).

**Table 3:** Codon preference analysis of chloroplast genome in *Impatiens*.

Species	Condon type number	Condon Total number	Codon type with Maximum number	Maximum number	Codon type with Minimum number	Minimum number	RSCU range	RSCU >1
*I. lateristachys*	64	50, 696	UUU(F)	2, 333	CGC(R)	220	0.43-1.99	35
*I.siculifer* var. *porphyrea*	64	50, 796	UUU(F)	2, 371	GCG(A)	231	0.50-2.04	37
*I. apalophylla*	64	50, 876	UUU(F)	2, 296	CGC(R)	226	0.44-1.96	37
*I. pritzelii*	64	50, 740	UUU(F)	2, 323	CGC(R)	220	0.43-2.12	35
*I. menghuochengensis*	64	50, 728	UUU(F)	2, 282	GCG(A)	230	0.47-2.04	35
*I. membranifolia*	64	50, 740	UUU(F)	2, 337	GCG(A)	224	0.48-1.96	35
*I. qingchengshanica*	64	50, 810	UUU(F)	2, 433	CGC(R)	234	0.44-2.00	35
*I. aquatilis*	64	50, 863	UUU(F)	2, 363	GCG(A)	224	0.47-2.00	36
*I. racemosa*	64	50, 594	UUU(F)	2, 321	GCG(A)	217	0.46-2.14	35

**Figure 4:**
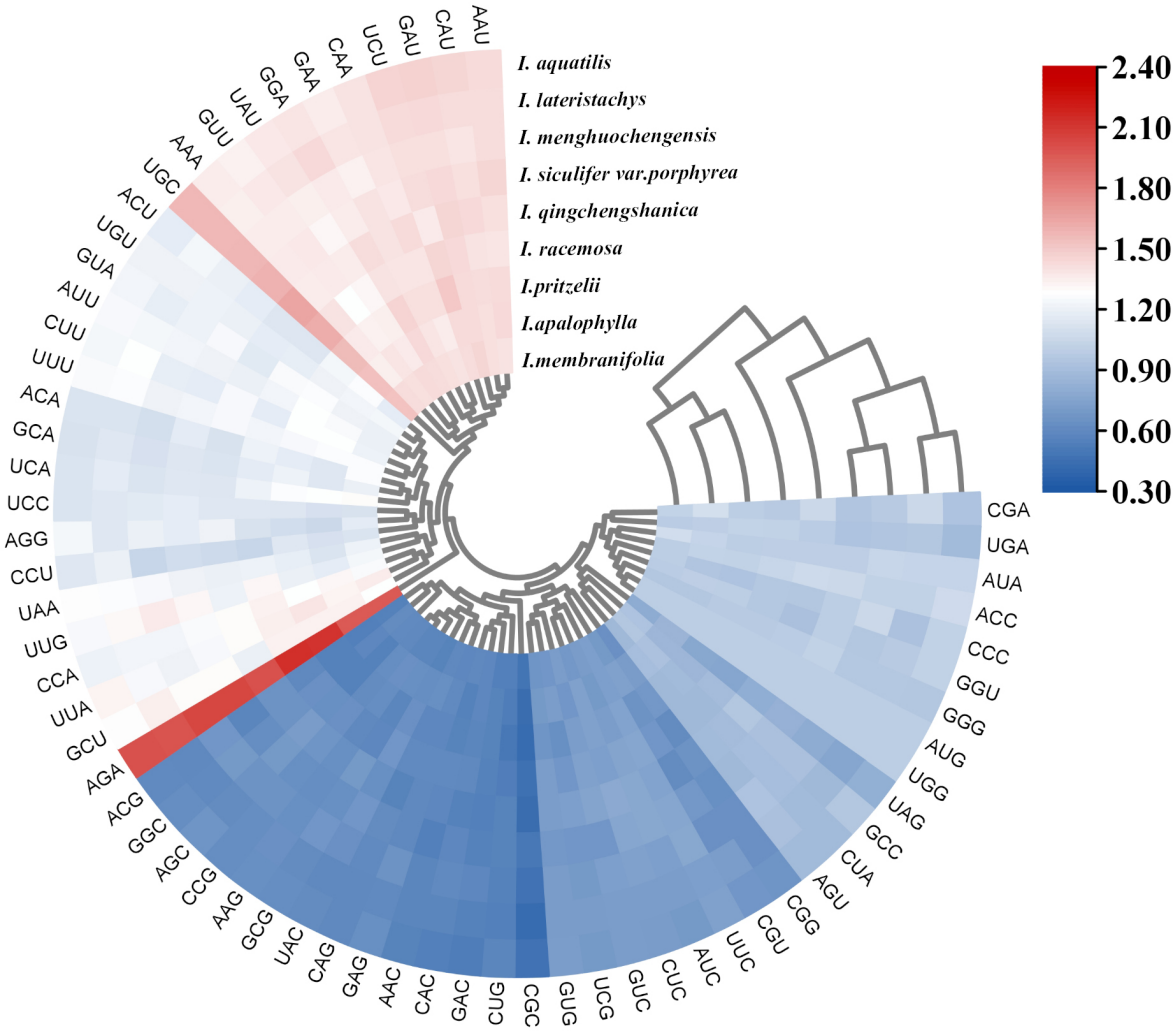
Cluster heatmap analysis of synonymous codons in the chloroplast genome of *Impatiens.* The RSCU values range from 0.3 to 2.4, corresponding to a color gradient from blue to red.

Among these 9 *Impatiens* species, 35-37 types of codons with an RSCU value ≥ 1.00 were identified, predominantly those ending with A/U, while those ending with C/G were relatively scarce. The arginine (Arg)-encoding codon AGA had the greatest RSCU score (Arg) (**Figure 4**). Additionally, the RSCU values of the chloroplast genomes predominantly ended with A/U, consistent with the characteristic low GC content of these genomes. The findings showed that the 9 *Impatiens* species shared a significant degree of similarity in codon usage and amino acid frequencies.

### 3.5 Comparative analysis of the IR boundary regions

**Figure 5:**
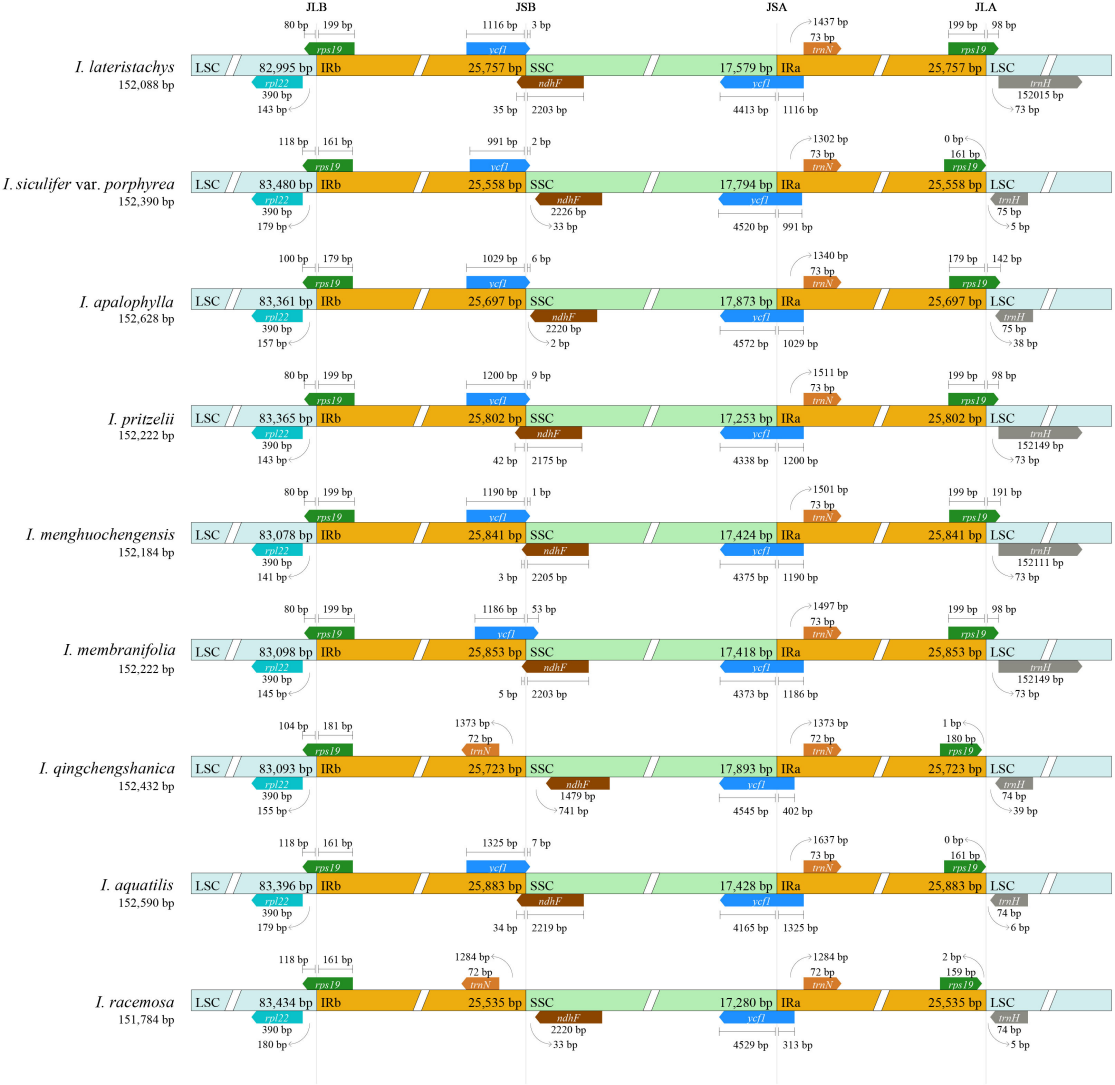
Compares the boundaries of the large single-copy region (LSC), small single-copy region (SSC), and inverted repeat region (IR) in the chloroplast genomes of 9 species. The distances between the gene ends and the border points are shown by the numbers above the gene features. JLB (IRb/LSC), JSA (SSC/IRa), JSB (IRb/SSC), and JLA (IRa/LSC) denote the junction sites between the quadripartite regions of the genome.

In this study, the chloroplast genomes of 9 *Impatiens* species were analyzed, focusing on the IR border regions. All genes and sequence lengths exhibited a tetrad structure and were highly conserved. However, differences were observed in the boundary regions, with contractions and expansions of the IR boundaries leading to variations in structure and size. The lengths of the IR regions in the 9 *Impatiens* species ranged from 25,535 bp (*I. racemosa*) to 25,883bp (*I. aquatilis*). The LSC region ranged from 80 to 118 bp, and the IR region overlap ranged from of 161 to 199bp. The *rps19* gene was located at the intersection of the LSC and IR sections in most species. The entire *rpl22* gene was located in the LSC region, near the IR boundary. In most *Impatiens* species, the *ndhF* gene was located at the boundary between the SSC and IR regions, with in half of the species, it was entirely within the SSC region. The *ycf1* gene spanned both the SSC and IR regions in all 9 *Impatiens* species, with lengths ranging from 4, 165 to 4,545 bp in the SSC region and 313 to 1,325 bp in the IR region. Copies of the *ycf1* gene were present in all species except *I. qingchengshanica*, and *I. racemosa*. The *trnN* gene was entirely located in the IR region in most species, while the *trnH* gene was located entirely in the LSC region. Gene contractions and expansions at the IR/SSC boundary varied among species (**Figure 5**).

### 3.6 Sequence divergence analysis of the chloroplast genomes of *Impatiens*

**Figure 6:**
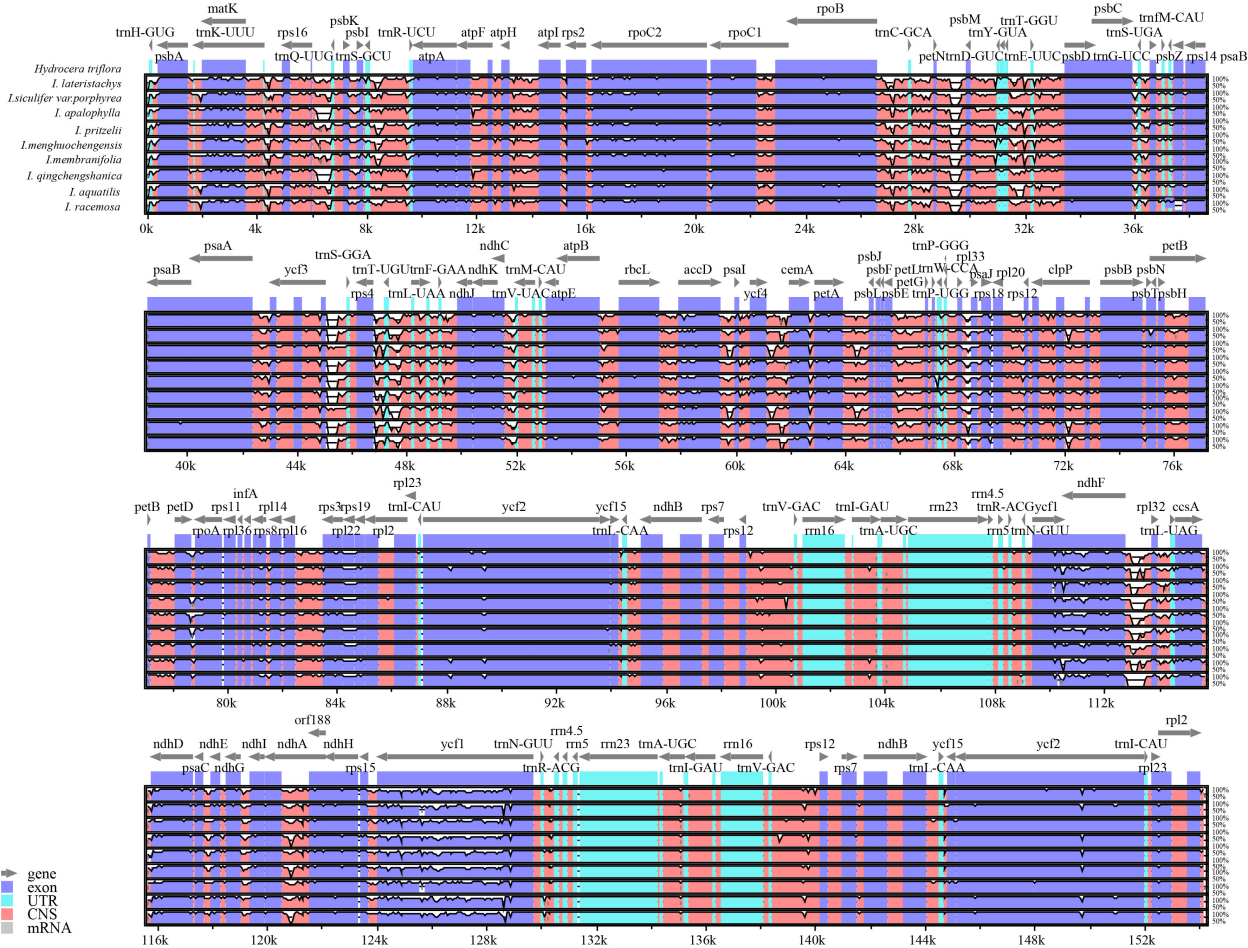
Compares the chloroplast genomes of 9 *Impatiens* species with that of *Hydrocera triflora* using the mVISTA method. The x-axis represents the positions within the chloroplast genome, and the y-axis represents the percentage of identity, ranging from 50% to 100%.

To investigate the differentiation of chloroplast genome sequences in *Hydrocera triflora* and other *Impatiens* species, highly variable regions were identified using the mVISTA software. Sequence homology across the entire chloroplast genome was analyzed using *Hydrocera triflora* as the reference genome, with results shown in the sequence homology plots (**Figure 6**). The findings showed a high degree of similarity between the chloroplast genomes of the 10 species, with strong conservation, high collinearity, and significant homology, reflecting a high degree of similarity. However, certain differences were observed, with varying mutation rates among the IR, SSC, and LSC regions, where the IR region was found to be more conserved. Coding regions showed higher conservation compared to non-coding regions. However, high divergence was observed in both intergenic spacers and coding genes, such as *matK*, *psbK*, *rps16*, *petN*, *trnC-GCA*, *rpoB*, *rps18*, *rpl33*, *ycf3*, *ndhE*, *ndhG*, *ycf1*, and *trnR-ACG*. Among the intergenic regions. The largest variations among intergenic regions were observed in *psbI-atpA*, *trnC-GCA-petN*, *ndhG-ndhI*, and *psbM-psbD*.

### 3.7 Analysis of mutation hotspots in the chloroplast genomes of *Impatiens*

**Figure 7:**
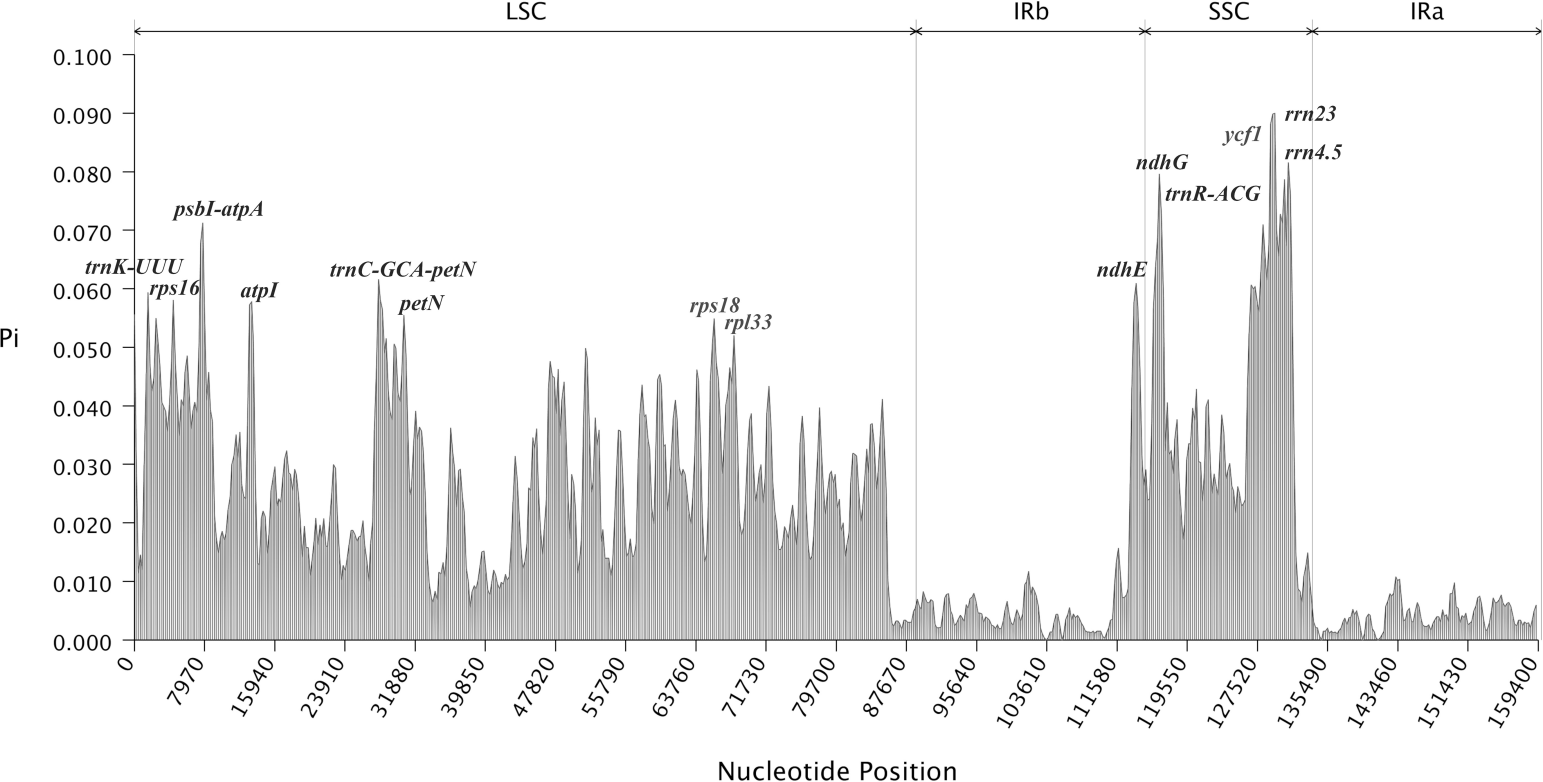
Mutational hotspot analysis. x-axis indicates regions of the chloroplast genome and y-axis indicates the nucleotide diversity of each region. Window size: 600 bp, step size: 200 bp.

Various hotspots were utilized to identify closely related species, providing crucial evidence for species identification through comparisons of gene distribution with the results of sliding window analysis. Nucleotide polymorphism analysis showed that sequence divergence in the LSC and SSC single-copy regions was significantly higher than in the inverted repeat region. The analysis indicated that nucleotide values in intergenic regions were higher than those in coding regions, suggesting greater divergence intergenic regions (**Figure 7**). The average nucleotide diversity across the 9 *Impatiens* species was 0.021856. The nucleotide diversity was highest in *rrn23* (0.09015) and *ndhG* (0.07973). Ten highly divergent hotspots were identified, including *trnK-UUU*, *psbI-atpA*, *atpI*, *trnC-GCA-petN*, and *rps18* in the LSC region, and *ndhE*, *ndhG*, *trnR-ACG*, *ycf1*, and *rrn23* in the SSC region. The IR region exhibited strong conservation, with no highly dispersed hotspots observed. Compared to the LSC region, the SSC region displayed more pronounced, highly dispersed hotspots, indicating greater variability. The hotspots identified by the mVISTA program were comparable to regions exhibiting the largest variations in Pi values. These distinct molecular markers could serve as valuable markers for phylogenetic analysis and species identification when combining results from DnaSP and mVISTA.

### 3.8 Analysis of selection pressure on chloroplast genome sequences in the *Impatiens*

**Figure 8:**
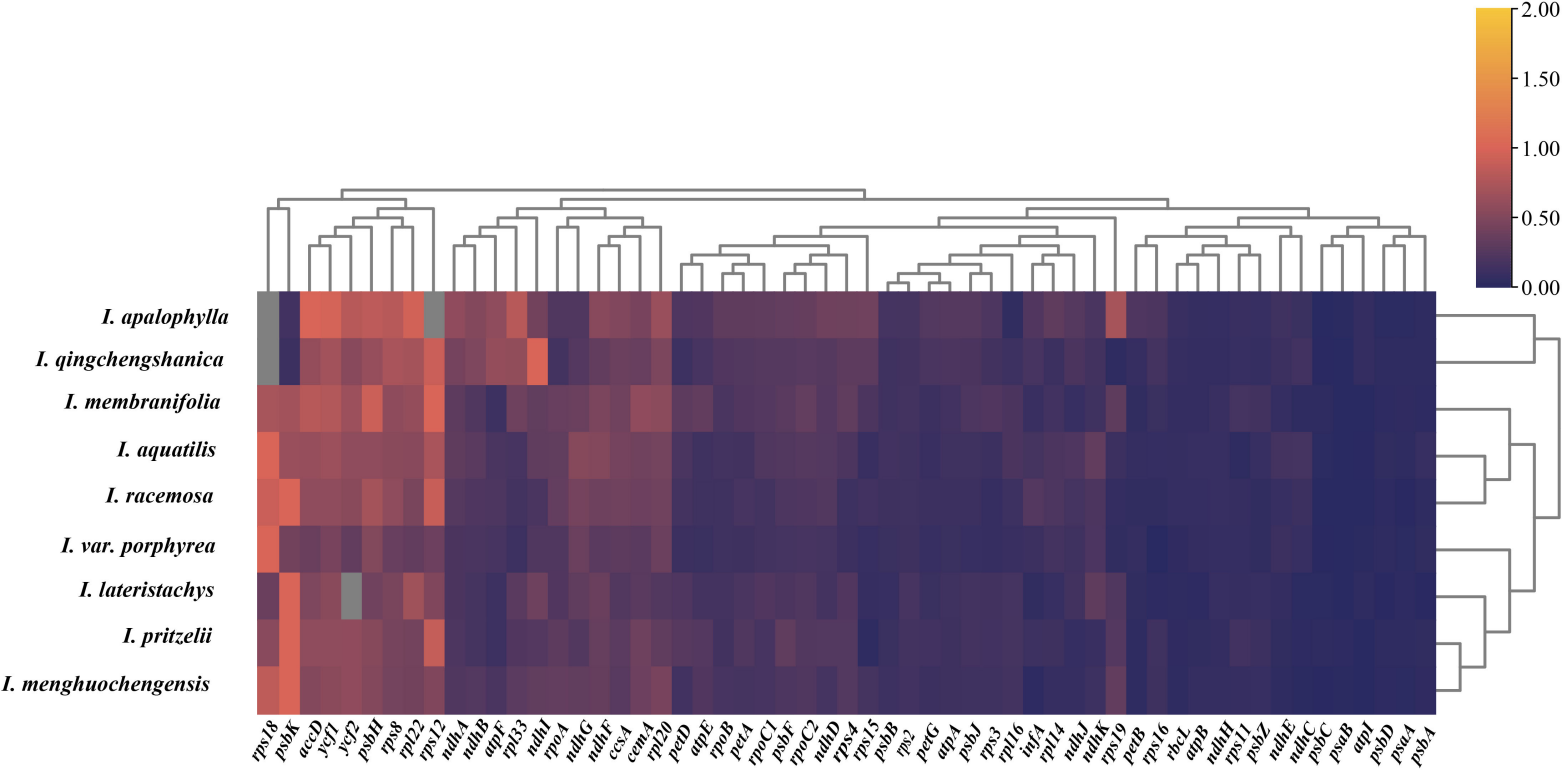
Selective pressure analysis results. A clustering heatmap of Ka/Ks values for the chloroplast genomes of 9 species with *Hydrocera triflora* as the reference species, the Ka/Ks values range from 0 to 2, corresponding to a color gradient from dark purple to orange.

To investigate the selective pressures acting on the chloroplast genome, we used *Hydrocera triflora* as a reference species and analyzed the selective and evolutionary differences among 9 species of *Impatiens*. We calculated the synonymous (Ks) and nonsynonymous (Ka) substitution rates, as well as the average Ka/Ks ratio (ω) for 74 protein-coding genes. We calculated the synonymous (Ks) and nonsynonymous (Ka) substitution rates, as well as the average Ka/Ks ratio (ω) for 74 protein-coding genes. Among these, 58 genes were selected for clustering heatmap analysis (**Figure 8**). Our results indicated that most genes were under purifying selection, with only a few genes showing evidence of positive selection. Notably, no genes exhibited neutral selection. Specifically, the *psbK* gene in *I. lateristachys*, *I. pritzelii*, *I. menghuochengensis*, and *I. racemosa*, the *rps18* gene in *I. siculifer* var. *porphyrea*, I. *menghuochengensis*, I. *aquatilis*, and *I. racemosa*, and the *rpl12* gene in *I. pritzelii* and *I. racemosa* all had Ka/Ks ratios greater than 1, suggesting that these genes were under positive selection.

### 3.9 Phylogenetic analysis of chloroplast genomes in *Impatiens*

This study was based on the complete chloroplast genome sequences of 24 plants from the Ericales order, including 21 *Impatiens* species, one *Hydrocera* species, and two outgroup species (one each from the Primulaceae and Actinidiaceae). A phylogenetic tree was constructed using maximum likelihood methods in IQtree, and the results were visualized and refined with FigTree, yielding a highly supported topology. These species have been extensively studied for their evolutionary relationships through morphology, palynology, and molecular markers, providing a robust foundation for our analysis. The selection of these species aims to illuminate the evolutionary history and taxonomic placement of *Impatiens* plants, validate previous findings, and elucidate their phylogenetic relationships through chloroplast genomic analysis.

**Figure 9:**
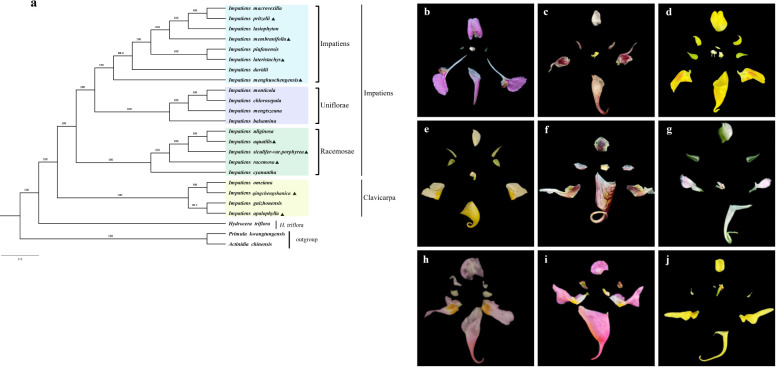
Phylogenetic tree was constructed based on the chloroplast genome sequences of 21 *Impatins* species and three other related species using the Maximum Likelihood (ML) method. **(a)**:Triangles stand for the species that our study has recently sequenced. and the colored blocks correspond to the different groups indicated. **(b-j)**: The Anatomical Structure of the Floral Parts of 9 *Impatiens* Species. **(b)**: *I. lateristachys*, **(c)**: *I. siculifer* var. *porphyrea*, **(d)**: *I. apalophylla*, **(e)**: *I. pritzelii*, **(f)**: *I. menghuochengensis*, **(g)**: *I. membranifolia*, **(h)**: *I. qingchengensis*, **(i)**: *I. aquatilis*, **(j)**: *I. racemosa*.

The evolutionary tree consisted of two primary branches. One branch included plants from the Balsaminaceae family, comprising 20 species of *Impatiens* and *Hydrocera triflora*, while the other branch included species from other families (**Figure 9**). Further analysis revealed that the Balsaminaceae family was divided into two distinct branches: the genera *Hydrocotyle* and *Impatiens*, consistent with previous classification studies. The genus *Impatiens* was further divided into two subgenera: *Clavicarpa* and *Impatiens*. *I. qingchengshanica* and *I. apalophylla*, along with *I. guizhouensis* and *I. omeiana*, form a group classified as the subgenus *Clavicarpa*, while the remaining *Impatiens* species are classified under the subgenus *Impatiens*. In the subgenus *Clavicarpa*, species such as *I. qingchengshanica* and *I. apalophylla* possess ovaries with four carpels, each containing one ovule. Their capsules are hammer-shaped, and the pollen germination grooves are trichotomous, showing a triangular view from the apex. The subgenus *Impatiens* was divided into three sections: sect. *Racemosae*, sect. *Uniflorae*, and sect. *Impatiens*. *I. aquatilis*, *I. racemosa*, and *I. siculifer* var. *porphyrea* cluster with *I. cyanantha* and *I. uliginosa*, characterized by ovaries with five carpels, linear capsules, racemose and many-flowered inflorescences, two laterally situated sepals, and oval seeds, classifying them in the sect. *Racemosae*. *I. chlorosepala* was closely related to *I. mengtszeana*, and both were classified in the sect. *Uniflorae*, characterized by ovaries with five carpels, spindle-shaped capsules, clustered inflorescences, and oval seeds. *I. menghuochengensis*, *I. lateristachys*, and *I. membranifolia* cluster with other *Impatiens* species in the sect. *Impatiens*, characterized by ovaries with five carpels, linear capsules, few-flowered racemose inflorescences, and oval seeds.

## 4 Discussion

### 4.1 Chloroplast genome structure

The circular structure of the chloroplast genomes of the 9 *Impatiens* species studied consists of a quadripartite structure, including two Inverted Repeat (IR) regions, one Large Single Copy (LSC) region, and one Small Single Copy (SSC) region. This structure is similar to that of other species in the Ericales, such as *Alniphyllum* (Ebenaceae), *Primula* (Primulaceae), and *Camellia* (Theaceae) (Han et al., 2017). It is also consistent with the chloroplast genome structures of other previously published *Impatiens* species (Luo et al., 2021).

Chloroplast genomes in most photosynthetic species range from 115 to 165 kb in size (Jansen et al., 2005). The chloroplast genome sizes in the *Impatiens* species studied ranged from 151, 784 bp (*I. racemosa*) to 152,628 bp (*I. apalophylla*), falling within the size range of published angiosperm chloroplast genomes (Dong et al., 2021; Li et al., 2024; Liu et al., 2024). Among the 9 *Impatiens* species, the largest variation in chloroplast genome size was 844 bp. The LSC region ranged from 82, 995 bp (*I. lateristachys*) to 83, 480 bp (*I. siculifer* var.*porphyrea*), the SSC region ranges from 17, 253 bp (*I. pritzelii*) to 17, 893 bp (*I. qingchengshanica*), and the IR region ranged from 25, 535 bp (*I. racemosa*) to 25, 883 bp (*I. aquatilis*). Comparative analysis indicated that the interspecific genomes within the genus *Impatiens* were relatively conserved, with size differences primarily arising from variations in the LSC, contractions and expansions of the IR region, as well as gene insertions and deletions. The chloroplast genomes of plants can contain between 63 and 209 genes, but most are concentrated in the range of 110 to 130 genes (Jansen et al., 2005). With gene functions and GC content in line with earlier research findings (Jansen et al., 2005), the chloroplast genomes of 9 *Impatiens* species were found to include between 108 and 117 genes (including 77 to 81 protein-coding genes, 28 to 32 tRNA genes, and 4 rRNA genes).

### 4.2 Repetitive sequence and simple sequence repeat analyses

SSRs, also known as microsatellite sequences in chloroplast genomes, are repetitive units consisting of one to six nucleotides, commonly found in eukaryotic cells. They are widely used in phylogenetic analyses, species identification, and genetic diversity assessments (Zhao et al., 2022). Genomic analyses of various chloroplasts have shown that indels and substitutions are likely induced by repetitive sequences (Gu et al., 2018). These sequences influence interspecies variations in copy numbers and play a crucial role in the stability and rearrangement of chloroplast genome sequences (Park et al., 2018). A total of 832 SSR sequences were identified in the cpDNA of 9 *Impatiens* species, including mononucleotides, dinucleotides, trinucleotides, and tetranucleotides. Pentanucleotides were not detected, while hexanucleotides (TAAGTA) were observed only in *I. pritzelii*. Previous studies on SSR motifs in eight Ericaceae species revealed hexanucleotide repeats in *Ardisia polysticta*, a member of the Primulaceae family (Li et al., 2018). This finding aligns with our results, which similarly demonstrate that not all SSR types were identified in every species. Previous studies on SSR motifs in eight Ericaceae species revealed hexanucleotide repeats in Ardisia polysticta, a member of the Primulaceae family (Li et al., 2018). This finding aligns with our results, which similarly demonstrate that not all SSR types were identified in every species. Mononucleotide repeats predominated, leading to a significantly lower GC content in the cpDNA of this genus compared to AT content. This pattern is consistent with findings in most plants, where A/T base repeats are the most abundant, as seen in other species (Li et al., 2022). Numerous cpSSR fragments were found in the chloroplast genomes of *Impatiens*, mainly composed of A/T mononucleotide repeat sequences, as well as polyadenine (polyA) and polythymidine (polyT). This high abundance of A/T bases is typical in plant chloroplast genomes (Ebert and Peakall, 2009). The development of SSR molecular markers provides offers valuable tools for further elucidating the phylogenetic and evolutionary relationships of *Impatiens* species.

### 4.3 Codon usage

Codon bias refers to the unequal usage of synonymous codons to encode the same amino acid across different organisms. This phenomenon has evolved over time due to various factors, including gene function, environmental selection, and gene expression levels. Species that are closely related exhibit similar codon usage patterns, offering valuable insights into interspecies evolution, exogenous gene expression, and genetic diversity (Wang et al., 2017). In this study, the 9 *Impatiens* species encoded between 50,594 (*I. racemosa*) and 50,876 (*I. apalophylla*) codons, with 35 to 37 codons showing an RSCU value of ≥ 1.00, mostly ending with A/U and a smaller number with C/G. This pattern aligns with the codon usage analyses in the chloroplast genomes of other higher plants (Gao et al., 2023). The GC content at the third codon position (GC3) plays a crucial role in influencing codon usage bias. Genomes with high GC content tend to favor codons enriched in G and C, while those with low GC content preferentially use codons rich in A and U (Jia et al., 2025). In this study, the average GC content of the chloroplast genome was approximately 37%, classifying it as low-GC. Therefore, codon usage in these chloroplast genomes predominantly favors codons with higher A and U content. This finding supports observations from previous studies. The genomes of the species studied exhibited a higher frequency of codons encoding leucine, consistent with previous reports (Yang et al., 2018; Guo et al., 2018; Wang et al., 2017). This study identified multiple codons encoding alanine (Ala), including GCA, GCT, GCC, and GCG, consistent with previous research (Sharp and Li, 1986). Previous studies have shown that codon usage bias is closely linked to factors such as mRNA stability and tRNA recognition efficiency (Hanson and Coller, 2018). In this study, we found that the frequently used codon AGA corresponds to tRNAs with higher abundance. This correspondence may affect mRNA stability by modulating tRNA recruitment and ribosomal translation. However, the precise molecular mechanisms and regulatory pathways underlying these relationships need further investigation for full elucidation. Given the highest degree of codon usage similarity among the 9 species, it is possible that these species encountered comparable environmental stresses in their biological niches.

### 4.4 IR expansion and contraction

The IR region contracts and expands at the boundaries of the LSC and SSC regions. These processes are key drivers of gene length variation. This occurs despite the generally high conservation of chloroplast genomes in terrestrial angiosperms (Zhu et al., 2016). Within chloroplast genomes, the IR region is generally regarded as the most conserved. However, variations in genome size, often caused by expansions and contractions in different plant lineages, make these regions useful for studying plant phylogenetic classifications (Wang et al., 2008). Events of contraction and expansion at the four boundary regions enhance our understanding of genome evolution and taxonomic hierarchies at or above the genus level. Aligning coding and non-coding sequences also aids in identifying mutation sites, offering essential data for studying interspecific and intrageneric phylogenetics and species evolution (Tang, 2017).

In this study of *Impatiens* species, genes at the IR/SC boundary underwent contraction and expansion, with the *rps19* gene at the junction of the LSC and IR regions showing duplication. Most other chloroplast genomes also exhibited this characteristic (Su et al., 2014; Lee et al., 2015). Previous comparative analyses of the inverted repeat (IR) regions among Ericaceae, Balsaminaceae, and other families have reported the duplication of the *rps19* gene only in Balsaminaceae species. Consistent with these findings, our study also identified the duplication of the *rps19* gene. The *ndhF* gene was located at the intersection of the SSC and IR sections, while the *rpl22* gene was entirely within the LSC region, close to the IR boundary. Previous studies have highlighted the crucial role of the *ycf1* gene in plant viability. In this study, the *ycf1* gene extended into the SSC region, varying in length across genomes, and spanning both the SSC and IR regions (Dong et al., 2015). Differences in the length and distribution of the *ycf1* gene, along with positional variations of the *rps19* and *ndhF* genes, contributed to length discrepancies within the *Impatiens* genus. Due to IR shrinkage, angiosperm chloroplast genomes often show variability in the number of duplicated genes, with duplication differences across species (Menezes et al., 2018). Copies of some pseudogenes, such as the *ycf1* gene, were retained at the boundaries, as seen in the *Withania somnifera* (Mehmood et al., 2020). These variations are crucial for understanding the evolution of chloroplast genomes and genomic structures (Kim K. et al., 2017). These analyses thus enhance our understanding of the genetic structure and evolutionary dynamics of *Impatiens* species.

### 4.5 Sequence divergence and mutational hotspots

Using *Hydrocera triflora* as a reference, mVISTA analysis revealed high sequence collinearity, low variation, and high segment sequence similarity. Notably, the LSC and SSC regions exhibited considerably more variance than the IR region, with non-coding regions showing significantly higher variation than coding regions. This may be related to selective pressures, where lower selective pressure led to structural variation, while higher selective pressure resulted in greater structural stability (Huang et al., 2024c; Zhang, 2022). Therefore, primers for species identification can be created based on polymorphic regions near these boundaries.

Nucleotide polymorphisms analysis is an indicator of the degree of polymorphism and variation in nucleotide sequences between species. Regions with high variability may serve as molecular markers for population genetics studies. The chloroplast genome contains a number of mutation hotspots that have been verified as possible molecular markers, laying the foundation for studying phylogeny and relationships among plants (Dong et al., 2021). Luo identified mutational hotspots in six *Impatiens* species, including *rps4-ndhJ, rpl32-ccsA*, *trnK-UUU-rps16*, *rpoB-petN*, *trnG-GCC*, *atpH-atpL*, *accD-psaI*, *ndhF*, and *ycf1* as potential molecular markers (Luo et al., 2021). In this study, 10 high-mutation hotspots were identified in the newly sequenced species: *trnK-UUU*, *psbI-atpA*, *atpI*, *trnC-GCA-petN*, *rps18*, *ndhE*, *ndhG*, *trnR-ACG*, *ycf1*, and *rrn23*, all sharing similar mutation characteristics. This study also found that the IR region was more conserved than the LSC and SSC regions, consistent with findings in other angiosperms (Lu et al., 2017; Wu et al., 2021). Divergence hotspots in the chloroplast genome have been widely used to determine the species of closely related plants (Dong et al., 2021; Bi et al., 2018). Therefore, we proposed that these ten highly variable regions could serve as DNA barcodes for *Impatiens* and be used in intra-species phylogeographic studies. External factors that influence a species’ evolutionary process and promote environmental adaption are known as selective pressure. When ω>1, advantageous mutations are selected, when ω =1, the species undergoes neutral selection, and when 0<ω<1, purifying selection occurs. A smaller ω indicates stronger negative selection pressure and greater conservation of the amino acid sequence (Fiz-Palacios et al., 2011). Analysis of selective pressure across 9 species revealed that most were under purifying selection, indicating a high level of conservation. Purifying selection was observed in most plants, suggesting a highly preserved evolutionary history (Huang et al., 2021). Purifying selection helped to prevent mutations (Wu et al., 2020), eliminating deleterious mutations and preserving conserved gene functions (Huang et al., 2021). In *Impatiens*, purifying selection may explain the high level of interspecific conservatism observed in the chloroplast genome. The adaptive evolution identified in this study may explain the observed diversity within the genus *Impatiens*, including habitat variation and morphological differences. Future research could further elucidate the environmental or functional drivers underlying the positive selection of these chloroplast genes, deepening our understanding of the adaptation and evolution of *Impatiens* species.

### 4.6 Phylogenetic analyses within Balsaminaceae species

Chloroplast genomes are maternally inherited and exhibit low rates of base substitution and structural rearrangements, making them valuable tools for studying phylogenetic relationships (Fang et al., 2021). The genus *Impatiens* includes a diverse range of species with complex and varied morphological characteristics, which pose significant challenges for phylogenetic analysis and identification. In this study, chloroplast genome sequences of 24 species were analyzed using maximum likelihood methods for phylogenetic analysis. The results indicated that the genus *Impatiens* was mainly divided into two evolutionary branches, consistent with prior studies (Janssens et al., 2012; Li et al., 2018). Based on the phylogenetic analysis of chloroplast genome sequences, this study supported the classification of the genus *Impatiens* into two subgenera (*Clavicarpa* and *Impatiens*) by Yu et al. (2016). The newly sequenced 9 species were categorized into different subgenera and further divided into three sections: sect. *Impatiens*, sect. *Racemosae*, and sect. *Uniflorae*. The study found that *I. qingchengshanica* and *I. apalophylla* were closely related to *I. omeiana* and *I. guizhouensis*, representing the basal lineage of *Impatiens*. A notable characteristic was the presence of three pollen apertures, which Lu considered to be a primitive pollen type (Lu, 1991), as this pollen type was found in the genus *Hydrocera* and subgenera *Clavicarpa*, aligning with the classifications by Zhang et al. (2023) and Xia (2020). *I. siculifer* var. *porphyrea* was considered as a variety of *I. siculifer*, sharing similar morphological characteristics. This study placed it in the sect. *Racemosae*, consistent with Yu et al. (2016) classifications based on morphology and molecular data. The sect. *Impatiens* included previously unpublished species *I. menghuochengensis* and *I. membranifolia*, as well as classified species,*I. pritzelii*, and *I. lateristachys*. Both *I. menghuochengensis* and *I. membranifolia* exhibit four pollen apertures and share morphological traits with the sect. *Impatiens*, a classification supported by chloroplast genomic data. Morphologically, *I. chlorosepala* resembled *I. mengtszeana*, and genomic analyses also revealed a close phylogenetic relationship between the two. In 2012, *I. lateristachys* was classified into the sect. *Fusicarpa* based on morphological taxonomy (Yu, 2012). However, it was reclassified into the sect. *Impatiens* in a 2016 revision (Yu et al., 2016), a classification supported by our results. Traditional morphological classification methods are often constrained by the similarity or diversity of morphological traits, which can obscure the accurate depiction of species’ taxonomic status. In contrast, this study leverages the sequence conservation of chloroplast genomes to provide robust molecular markers for phylogenetic analysis, thereby more precisely elucidating the taxonomic position of *I. lateristachys*. In this study, *I. pritzelii* was closely related to *I. macrovexilla* and *I. membranifolia*, with a high support value (BS=100). According to Yu et al. (2016), *I. pritzelii* was classified in the *Clavicarpa* subgenus, which was inconsistent with our results. We speculated that the chloroplast genome length of *I. pritzelii* was similar to that of *I. membranifolia*, with only minor differences in IR expansion and contraction, further supporting their phylogenetic relationship from a genomic perspective. This indicated that the classification results are integrative, combining both macroscopic morphological features and molecular data. Using the entire chloroplast genome sequences, our classification aligns with Yu et al. (2016) classification, demonstrating a high congruence between genomic and morphological classifications in phylogenetic relationships. This study employed complete chloroplast genome sequences for classification, which showed a high consistency with Yu et al. (2016) classification. This congruence highlights the strong alignment between genomic and morphological classifications in elucidating phylogenetic relationships.

The chloroplast genomes used in this study provided extensive genetic information and high-resolution evolutionary signals, facilitating precise phylogenetic analysis of *Impatiens* species. Their maternal inheritance and structural stability make them ideal for clarifying kinship among *Impatiens* species, while their moderate evolutionary rate effectively reveals evolutionary events between these species. Chloroplast genome analysis identified positively selected genes such as *psbK*, *rps18*, and rpl12, potentially linked to the adaptive evolution of *Impatiens* species in diverse environments. Additionally, the conservation and diversity of chloroplast genomes offer essential criteria for identifying and conserving *Impatiens* species, further advancing taxonomic and ecological studies in this genus.

## 5 Conclusion

This study examines the chloroplast genomes of 9 *Impatiens* species, revealing high similarity in structure, size, GC content, gene number, and function, highlighting the conserved nature of these genomes. We identified expansions and contractions within the inverted repeat (IR) regions and found ten divergent areas that could serve as markers for phylogenetic classification and species identification. Most species showed purifying selection, leading to highly conserved genomic sequences. The phylogenetic tree, constructed using the maximum likelihood method, supports the classification and evolutionary relationships of *Impatiens* species. These genomic data provide a solid foundation for understanding the evolutionary dynamics within the *Impatiens* genus. Future research should integrate morphological traits, molecular studies, and genomic analyses to address classification and evolutionary questions in *Impatiens*.

## Data Availability

New sequenced and other published chloroplast genome 716 sequences can be found in GenBank (https://www.ncbi.nlm.nih.gov/genbank/) with the accession numbers present in **Supplementary Table S1**.
