# Supplementary material for: Phylogenetic analysis of nine Impatiens species from subgenus Clavicarpa and subgenus Impatiens (Sect. Impatiens and Sect. Racemosae) based on chloroplast genomes
Source: Front Plant Sci. 2025 Mar 27;16:1541320. doi: 10.3389/fpls.2025.1541320 (PMC11983548; doi:10.3389/fpls.2025.1541320)
Supplement: Supplementary file 1 [file DataSheet1.docx]

Table S1 Information on Collection Sites of 9 *Impatiens* Species

| **Species** | **Collection Site** | **Distribution Elevation/m** | **Latitude (N)** | **Longitude (E)** | **Habitat Overview** | **Accession** |
| --- | --- | --- | --- | --- | --- | --- |
| *I. lateristachys* | Jinhekou District, Leshan City, Sichuan Province, China | 1911.8 | 29°22'98" | 103°1'10" | Forest Edge | PQ612862 |
| *I.siculifer* var.*porphyrea* | Yanling County, Zhuzhou City, Hunan Province, China | 781.4 | 26°33′34′′ | 114°4′42′′ | Beside the stream | PQ612866 |
| *I. apalophylla* | Jinfo Mountain, Nanchuan County, Sichuan Province, China | 2238.2 | 28°31′ | 107°27′ | rock crevices | PQ612860 |
| *I. pritzelii* | Enshi Tujia and Miao Autonomous Prefecture, Hubei Province, China | 794 | 30°29′52′′ | 109°47′96′′ | shady wet places | PQ612865 |
| *I.menghuochengensis* | Shimian County, Sichuan Province, China | 2592.3 | 28°53'64" | 102°20'66" | Forest Edge Ditch | PQ612864 |
| *I.membranifolia* | Qingchuan County, Sichuan Province | 1419.9 | 32°34'37" | 104 10'75" | Next to Highway | PQ612863 |
| *I. qingchengshanica* | Mount Emei, Sichuan Province, China | 1492 | 29°16′ | 103°10′ | Mountainside | PQ619433 |
| *I. aquatilis* | Mengzi, Yunnan Province, China | 1455 | 23°60′00′′ | 107°12′43′′ | Underwater | PQ612861 |
| *I. racemosa* | Xinlong, Cizhuba Village, Malipo Town, Malipo County, Yunnan Province, China | 2163 | 23°7′ | 104°47′ | The grass beside the ditch | PQ619434 |

Table S2 Chloroplast genome gene information of *I. siculifer* var.*porphyrea*

| **Classification of genes** | **Subclassification of genes based on functions** | **Gene name** | | | | | **Total numbers** |
| --- | --- | --- | --- | --- | --- | --- | --- |
| Photosynthesis related genes | PhotosystemⅠ | *psaA* | *psaB* | *psaC* | *psaI* | *psaJ* | 5 |
|  | PhotosystemⅡ | *psbA* | *psbB* | *psbC* | *psbD* | *psbE* | 14 |
|  |  | *psbF* | *psbH* | *psbI* | *psbJ* | *psbk* |  |
|  |  | *psbL* | *psbM* | *psbT* | *psbZ* |  |  |
|  | Cytochrome  b/f compelx | *petA* | *petB** | *petD** | *petG* | *petL* | 6 |
|  |  | *petN* |  |  |  |  |  |
|  | ATP synthase | *atpA* | *atpB* | *atpE* | *atpF**(2) | *atpH* | 6 |
|  |  | *atpI* |  |  |  |  |  |
|  | NADPH dehydrogenase | *ndhA** | *ndhB**(2) | *ndhC* | *ndhD* | *ndhE* | 11 |
|  |  | *ndhF* | *ndhG* | *ndhH* | *ndhI* | *ndhJ* |  |
|  |  | *ndhK*(2) |  |  |  |  |  |
|  | Rubisco | *rbcl* |  |  |  |  | 1 |
| Self-replication | Transcription | *rpoA* | *rpoB* | *rpoC1**(2) | *rpoC2* |  | 4 |
|  | Small subunit  of ribosome | *rps11* | *rps12***(2) | *rps14* | *rps15* | *rps16^*^* | 12 |
|  |  | *rps18* | *rps19* | *rps2* | *rps3* | *rps4* |  |
|  |  | (2) | *rps8* |  |  |  |  |
|  | Large subunit  of ribosome | *rpl2*(*4) | *rpl14* | *rpl16** | *rpl20* | *rpl22* | 9 |
|  |  | *rpl23（2）* | *rpl32* | *rpl33* | *rpl36* |  |  |
|  | Translational initiation factor | *infA* |  |  |  |  | 1 |
|  | Ribosomal RNA | *rrn4.5*(2) | *rrn5*(2) | *rrn16*(4) | *rrn23*(4) |  | 4 |
|  | Transfer RNA | *trnA-UGC**(2) | *trnC-GCA* | *trnD-GUC* | *trnE-UUC* | *trnF-GAA* | 30 |
|  |  | *trnG-GCC* | *trnG-UCC** | *trnH-GUG* | *trnI-CAU*(2) | *trnI-GAU**(4) |  |
|  |  | *trnK-UUU** | *trnL-CAA*(2) | *trnL-UAA** | *trnL-UAG* | *trnM-CAU* |  |
|  |  | *trnN-GUU*(4) | *trnP-UGG* | *trnQ-UUG* | *trnR-ACG*(2) | *trnR-UCU* |  |
|  |  | *trnS-GCU* | *trnS-GGA* | *trnS-UGA* | *trnT-GGU* | *trnT-UGU* |  |
|  |  | *trnV-GAC*(2) | *trnV-UAC** | *trnW-CCA* | *trnY-GUA* | *trnfM-CAU* |  |
| Other genes | Cytochrome c synthesis | *ccsA* |  |  |  |  | 1 |
|  | RNA processing | *matK* |  |  |  |  | 1 |
|  | Carbon metabolism | *cemA* |  |  |  |  | 1 |
|  | Fatty acid synthesis | *accD* |  |  |  |  | 1 |
|  | proteolysis | *clpP*** |  |  |  |  | 1 |
|  | Other | *pafI***(2) | *pafII* | *pbf1* |  |  | 3 |
| Genes of unknown function | | *ycf1*(2) | *ycf2*(2) | *ycf15*(2) |  |  | 3 |
| Total genes | | | | | | | 114 |

Note: Gene*: Gene with one introns; Gene**: Gene with two introns; Gene(2): Number of copies of multi-copy genes.

Table S3 Chloroplast genome gene information of *I. apalophylla*

| **Classification of genes** | **Subclassification of genes based on functions** | **Gene name** | | | | | **Total numbers** |
| --- | --- | --- | --- | --- | --- | --- | --- |
| Photosynthesis related genes | PhotosystemⅠ | *psaA* | *psaB* | *psaC* | *psaI* | *psaJ* | 5 |
|  | PhotosystemⅡ | *psbA* | *psbB* | *psbC*(2) | *psbD* | *psbE* | 14 |
|  |  | *psbF* | *psbH* | *psbI* | *psbJ* | *psbk* |  |
|  |  | *psbL* | *psbM* | *psbT* | *psbZ* |  |  |
|  | Cytochrome  b/f compelx | *petA* | *petB** | *petD** | *petG* | *petL* | 6 |
|  |  | *petN* |  |  |  |  |  |
|  | ATP synthase | *atpA* | *atpB* | *atpE* | *atpF**(2) | *atpH* | 6 |
|  |  | *atpI* |  |  |  |  |  |
|  | NADPH dehydrogenase | *ndhA**(2) | *ndhB**(2) | *ndhC* | *ndhD* | *ndhE* | 11 |
|  |  | *ndhF* | *ndhG* | *ndhH* | *ndhI* | *ndhJ* |  |
|  |  | *ndhK* |  |  |  |  |  |
|  | Rubisco | *rbcL* |  |  |  |  | 1 |
| Self-replication | Transcription | *rpoA* | *rpoB* | *rpoC1**(2) | *rpoC2* |  | 4 |
|  | Small subunit  of ribosome | *rps11* | *rps12***(2) | *rps14* | *rps15* | *rps16**(2) | 12 |
|  |  | *rps18* | *rps19*(2) | *rps2* | *rps3* | *rps4* |  |
|  |  | *rps7*(2) | *rps8* |  |  |  |  |
|  | Large subunit  of ribosome | *rpl2**(4) | *rpl14* | *rpl16** | *rpl20* | *rpl22* | 9 |
|  |  | *rpl23*(2) | *rpl32* | *rpl33* | *rpl36* |  |  |
|  | Translational initiation factor | *infA* |  |  |  |  | 1 |
|  | Ribosomal RNA | *rrn4.5*(2) | *rrn5*(2) | *rrn16*(4) | *rrn23*(4) |  | 4 |
|  | Transfer RNA | *trnA-UGC**(2) | *trnC-GCA* | *trnD-GUC* | *trnE-UUC* | *trnF-GAA* | 30 |
|  |  | *trnG-GCC* | *trnG-UCC** | *trnH-GUG*(2) | *trnI-CAU*(2) | *trnI-GAU**(4) |  |
|  |  | *trnK-UUU**(2) | *trnL-CAA*(2) | *trnL-UAA**(2) | *trnL-UAG* | *trnM-CAU*(2) |  |
|  |  | *trnN-GUU*(4) | *trnP-UGG* | *trnQ-UUG* | *trnR-ACG*(2) | *trnR-UCU* |  |
|  |  | *trnS-GCU* | *trnS-GGA* | *trnS-UGA* | *trnT-GGU* | *trnT-UGU* |  |
|  |  | *trnV-GAC*(2) | *trnV-UAC** | *trnW-CCA* | *trnY-GUA* | *trnfM-CAU* |  |
| Other genes | Cytochrome c  Synthesis | *ccsA* |  |  |  |  | 1 |
|  | RNA processing | *matK* |  |  |  |  | 1 |
|  | Carbon metabolism | *cemA* |  |  |  |  | 1 |
|  | Fatty acid synthesis | *accD* |  |  |  |  | 1 |
|  | proteolysis | *clpP1***(2) |  |  |  |  | 1 |
|  | Other | *pafI***(2) | *pafII* | *pbf1* |  |  | 3 |
| Genes of unknown function | | *ycf1*(2) | *ycf2*(2) | *ycf15*(2) | *orf188* |  | 4 |
| Total genes | | | | | | | 115 |

Note: Gene*: Gene with one introns; Gene**: Gene with two introns; Gene(2): Number of copies of multi-copy genes.

Table S4 Chloroplast genome gene information of *I. pritzelii*

| **Classification of genes** | **Subclassification of genes based on functions** | **Gene name** | | | | | **Total numbers** |
| --- | --- | --- | --- | --- | --- | --- | --- |
| Photosynthesis related genes | PhotosystemⅠ | *psaA* | *psaB* | *psaC* | *psaI* | *psaJ* | 5 |
|  | PhotosystemⅡ | *psbA* | *psbB* | *psbC*(2) | *psbD* | *psbE* | 14 |
|  |  | *psbF* | *psbH* | *psbI* | *psbJ* | *psbk* |  |
|  |  | *psbL* | *psbM* | *psbT* | *psbZ* |  |  |
|  | Cytochrome  b/f compelx | *petA* | *petB** | *petD** | *petG* | *petL* | 6 |
|  |  | *petN* |  |  |  |  |  |
|  | ATP synthase | *atpA* | *atpB* | *atpE* | *atpF**(2) | *atpH* | 6 |
|  |  | *atpI* |  |  |  |  |  |
|  | NADPH dehydrogenase | *ndhA**(2) | *ndhB**(2) | *ndhC* | *ndhD* | *ndhE* | 11 |
|  |  | *ndhF* | *ndhG* | *ndhH* | *ndhI* | *ndhJ* |  |
|  |  | *ndhK* |  |  |  |  |  |
|  | Rubisco | *rbcL* |  |  |  |  | 1 |
| Self-replication | Transcription | *rpoA* | *rpoB* | *rpoC1**(2) | *rpoC2* |  | 4 |
|  | Small subunit  of ribosome | *rps11* | *rps12***(2) | *rps14* | *rps15* | *rps16**(2) | 12 |
|  |  | *rps18* | *rps19*(2) | *rps2* | *rps3* | *rps4* |  |
|  |  | *rps7*(2) | *rps8* |  |  |  |  |
|  | Large subunit  of ribosome | *rpl2**(2) | *rpl14* | *rpl16** | *rpl20* | *rpl22* | 9 |
|  |  | *rpl23*(2) | *rpl32* | *rpl33* | *rpl36* |  |  |
|  | Translational initiation factor | *infA* |  |  |  |  | 1 |
|  | Ribosomal RNA | *rrn4.5*(2) | *rrn5*(2) | *rrn16*(4) | *rrn23*(4) |  | 4 |
|  | Transfer RNA | *trnA-UGC**(2) | *trnC-GCA* | *trnD-GUC* | *trnE-UUC* | *trnF-GAA* | 30 |
|  |  | *trnG-GCC* | *trnG-UCC** | *trnH-GUG* | *trnI-CAU*(2) | *trnI-GAU**(4) |  |
|  |  | *trnK-UUU** | *trnL-CAA*(2) | *trnL-UAA**(2) | *trnL-UAG* | *trnM-CAU* |  |
|  |  | *trnN-GUU*(4) | *trnP-UGG* | *trnQ-UUG* | *trnR-ACG*(2) | *trnR-UCU* |  |
|  |  | *trnS-GCU* | *trnS-GGA* | *trnS-UGA* | *trnT-GGU* | *trnT-UGU* |  |
|  |  | *trnV-GAC*(2) | *trnV-UAC** | *trnW-CCA* | *trnY-GUA* | *trnfM-CAU* |  |
| Other genes | Cytochrome c synthesis | *ccsA* |  |  |  |  | 1 |
|  | RNA processing | *matK* |  |  |  |  | 1 |
|  | Carbon metabolism | *cemA* |  |  |  |  | 1 |
|  | Fatty acid synthesis | *accD* |  |  |  |  | 1 |
|  | proteolysis | *clpP1*** |  |  |  |  | 1 |
|  | Other | *pafI***(2) | *pafII* | *pbf1* |  |  | 3 |
| Genes of unknown function | | *ycf1*(2) | *ycf2*(2) |  |  |  | 4 |
| Total genes | | | | | | | 113 |

Note: Gene*: Gene with one introns; Gene**: Gene with two introns; Gene(2): Number of copies of multi-copy genes.

Table S5 Chloroplast genome gene information of *I.menghuochengensis*

| **Classification of genes** | **Subclassification of genes based on functions** | **Gene name** | | | | | **Total numbers** |
| --- | --- | --- | --- | --- | --- | --- | --- |
| Photosynthesis related genes | PhotosystemⅠ | *psaA* | *psaB* | *psaC* | *psaI* | *psaJ* | 5 |
|  | PhotosystemⅡ | *psbA* | *psbB* | *psbC*(2) | *psbD* | *psbE* | 14 |
|  |  | *psbF* | *psbH* | *psbI* | *psbJ* | *psbk* |  |
|  |  | *psbL* | *psbM* | *psbT* | *psbZ* |  |  |
|  | Cytochrome  b/f compelx | *petA* | *petB** | *petD** | *petG* | *petL* | 6 |
|  |  | *petN* |  |  |  |  |  |
|  | ATP synthase | *atpA* | *atpB* | *atpE* | *atpF**(2) | *atpH* | 6 |
|  |  | *atpI* |  |  |  |  |  |
|  | NADPH dehydrogenase | *ndhA**(2) | *ndhB**(2) | *ndhC* | *ndhD* | *ndhE* | 11 |
|  |  | *ndhF* | *ndhG* | *ndhH* | *ndhI* | *ndhJ* |  |
|  |  | *ndhK* |  |  |  |  |  |
|  | Rubisco | *rbcL* |  |  |  |  | 1 |
| Self-replication | Transcription | *rpoA* | *rpoB* | *rpoC1**(2) | *rpoC2* |  | 4 |
|  | Small subunit  of ribosome | *rps11* | *rps12***(2) | *rps14* | *rps15* | *rps16** | 12 |
|  |  | *rps18* | *rps19*(2) | *rps2* | *rps3* | *rps4* |  |
|  |  | *rps7*(2) | *rps8* |  |  |  |  |
|  | Large subunit  of ribosome | *rpl2**(4) | *rpl14* | *rpl16** | *rpl20* | *rpl22* | 9 |
|  |  | *rpl23*(2) | *rpl32* | *rpl33* | *rpl36* |  |  |
|  | Translational initiation factor | *infA* |  |  |  |  | 1 |
|  | Ribosomal RNA | *rrn4.5*(2) | *rrn5*(2) | *rrn16*(4) | *rrn23*(4) |  | 4 |
|  | Transfer RNA | *trnA-UGC**(2) | *trnC-GCA* | *trnD-GUC* | *trnE-UUC* | *trnF-GAA* | 30 |
|  |  | *trnG-GCC* | *trnG-UCC** | *trnH-GUG* | *trnI-CAU*(2) | *trnI-GAU**(4) |  |
|  |  | *trnK-UUU** | *trnL-CAA*(2) | *trnL-UAA** | *trnL-UAG* | *trnM-CAU* |  |
|  |  | *trnN-GUU*(4) | *trnP-UGG* | *trnQ-UUG* | *trnR-ACG*(2) | *trnR-UCU* |  |
|  |  | *trnS-GCU* | *trnS-GGA* | *trnS-UGA* | *trnT-GGU* | *trnT-UGU* |  |
|  |  | *trnV-GAC*(2) | *trnV-UAC** | *trnW-CCA* | *trnY-GUA* | *trnfM-CAU* |  |
| Other genes | Cytochrome c synthesis | *ccsA* |  |  |  |  | 1 |
|  | RNA processing | *matK* |  |  |  |  | 1 |
|  | Carbon metabolism | *cemA* |  |  |  |  | 1 |
|  | Fatty acid synthesis | *accD* |  |  |  |  | 1 |
|  | proteolysis | *clpP1*** |  |  |  |  | 1 |
|  | Other | *pafI***(2) | *pafII* | *pbf1* |  |  | 3 |
| Genes of unknown function | | *ycf1*(2) | *ycf2*(2) |  |  |  | 4 |
| Total genes | | | | | | | 113 |

Note: Gene*: Gene with one introns; Gene**: Gene with two introns; Gene(2): Number of copies of multi-copy genes.

Table S6 Chloroplast genome gene information of *I.membranifolia*

| **Classification of genes** | **Subclassification of genes based on functions** | **Gene name** | | | | | **Total numbers** |
| --- | --- | --- | --- | --- | --- | --- | --- |
| Photosynthesis related genes | PhotosystemⅠ | *psaA* | *psaB* | *psaC* | *psaI* | *psaJ* | 5 |
|  | PhotosystemⅡ | *psbA* | *psbB* | *psbC*(2) | *psbD* | *psbE* | 14 |
|  |  | *psbF* | *psbH* | *psbI* | *psbJ* | *psbk* |  |
|  |  | *psbL* | *psbM* | *psbT* | *psbZ* |  |  |
|  | Cytochrome  b/f compelx | *petA* | *petB** | *petD** | *petG* | *petL* | 6 |
|  |  | *petN* |  |  |  |  |  |
|  | ATP synthase | *atpA* | *atpB* | *atpE* | *atpF**(2) | *atpH* | 6 |
|  |  | *atpI* |  |  |  |  |  |
|  | NADPH dehydrogenase | *ndhA**(2) | *ndhB**(2) | *ndhC* | *ndhD* | *ndhE* | 11 |
|  |  | *ndhF* | *ndhG* | *ndhH* | *ndhI* | *ndhJ* |  |
|  |  | *ndhK* |  |  |  |  |  |
|  | Rubisco | *rbcL* |  |  |  |  | 1 |
| Self-replication | Transcription | *rpoA* | *rpoB* | *rpoC1**(2) | *rpoC2* |  | 4 |
|  | Small subunit  of ribosome | *rps11* | *rps12***(2) | *rps14* | *rps15* | *rps16** | 12 |
|  |  | *rps18* | *rps19*(2) | *rps2* | *rps3* | *rps4* |  |
|  |  | *rps7*(2) | *rps8* |  |  |  |  |
|  | Large subunit  of ribosome | *rpl2**(4) | *rpl14* | *rpl16** | *rpl20* | *rpl22* | 9 |
|  |  | *rpl23*(2) | *rpl32* | *rpl33* | *rpl36* |  |  |
|  | Translational initiation factor | *infA* |  |  |  |  | 1 |
|  | Ribosomal RNA | *rrn4.5*(2) | *rrn5*(2) | *rrn16*(4) | *rrn23*(4) |  | 4 |
|  | Transfer RNA | *trnA-UGC**(2) | *trnC-GCA* | *trnD-GUC* | *trnE-UUC* | *trnF-GAA* | 30 |
|  |  | *trnG-GCC* | *trnG-UCC** | *trnH-GUG* | *trnI-CAU*(2) | *trnI-GAU**(4) |  |
|  |  | *trnK-UUU** | *trnL-CAA*(2) | *trnL-UAA** | *trnL-UAG* | *trnM-CAU* |  |
|  |  | *trnN-GUU*(4) | *trnP-UGG* | *trnQ-UUG* | *trnR-ACG*(2) | *trnR-UCU* |  |
|  |  | *trnS-GCU* | *trnS-GGA* | *trnS-UGA* | *trnT-GGU* | *trnT-UGU* |  |
|  |  | *trnV-GAC*(2) | *trnV-UAC** | *trnW-CCA* | *trnY-GUA* | *trnfM-CAU* |  |
| Other genes | Cytochrome c synthesis | *ccsA* |  |  |  |  | 1 |
|  | RNA processing | *matK* |  |  |  |  | 1 |
|  | Carbon metabolism | *cemA* |  |  |  |  | 1 |
|  | Fatty acid synthesis | *accD* |  |  |  |  | 1 |
|  | proteolysis | *clpP1*** |  |  |  |  | 1 |
|  | Other | *pafI***(2) | *pafII* | *pbf1* |  |  | 3 |
| Genes of unknown function | | *ycf1*(2) | *ycf2*(2) |  |  |  | 4 |
| Total genes | | | | | | | 113 |

Note: Gene*: Gene with one introns; Gene**: Gene with two introns; Gene(2): Number of copies of multi-copy genes.

Table S7 Chloroplast genome gene information of *I.qingchengshanica*

| **Classification of genes** | **Subclassification of genes based on functions** | **Gene name** | | | | | **Total numbers** |
| --- | --- | --- | --- | --- | --- | --- | --- |
| Photosynthesis related genes | PhotosystemⅠ | *psaA* | *psaB* | *psaC* | *psaI* | *psaJ* | 5 |
|  | PhotosystemⅡ | *psbA* | *psbB* | *psbC*(2) | *psbD* | *psbE* | 15 |
|  |  | *psbF* | *psbH* | *psbI* | *psbJ* | *psbk* |  |
|  |  | *psbL* | *psbM* | *psbN* | *psbT* | *psbZ* |  |
|  | Cytochrome  b/f compelx | *petA* | *petB** | *petD** | *petG* | *petL* | 6 |
|  |  | *petN* |  |  |  |  |  |
|  | ATP synthase | *atpA* | *atpB* | *atpE* | *atpF**(2) | *atpH* | 6 |
|  |  | *atpI* |  |  |  |  |  |
|  | NADPH dehydrogenase | *ndhA**(2) | *ndhB**(2) | *ndhC* | *ndhD* | *ndhE* | 11 |
|  |  | *ndhF* | *ndhG* | *ndhH* | *ndhI* | *ndhJ* |  |
|  |  | *ndhK* |  |  |  |  |  |
|  | Rubisco | *rbcL* |  |  |  |  | 1 |
| Self-replication | Transcription | *rpoA* | *rpoB* | *rpoC1**(2) | *rpoC2* |  | 4 |
|  | Small subunit  of ribosome | *rps11* | *rps12***(2) | *rps14* | *rps15* | *rps16** | 12 |
|  |  | *rps18* | *rps19*(2) | *rps2* | *rps3* | *rps4* |  |
|  |  | *rps7*(2) | *rps8* |  |  |  |  |
|  | Large subunit  of ribosome | *rpl2*（4）* | *rpl14* | *rpl16** | *rpl20* | *rpl22* | 8 |
|  |  | *rpl23（2）* | *rpl33* | *rpl36* |  |  |  |
|  | Translational initiation factor | *infA* |  |  |  |  | 1 |
|  | Ribosomal RNA | *rrn4.5（2）* | *rrn5（2）* | *rrn16（4）* | *rrn23（4）* |  | 4 |
|  | Transfer RNA | *trnA-UGC**(2) | *trnC-GCA* | *trnD-GUC* | *trnE-UUC**(2) | *trnF-GAA* | 27 |
|  |  | *trnG-GCC* | *trnH-GUG* | *trnK-UUU** | *trnL-CAA*(2) | *trnL-UAA** |  |
|  |  | *trnL-UAG* | *trnM-CAU*(4) | *trnN-GUU*(2) | *trnP-UGG* | *trnQ-UUG* |  |
|  |  | *trnR-ACG*(2) | *trnR-UCU* | *trnS-GCU* | *trnS-GGA* | *trnS-UGA* |  |
|  |  | *trnT-GGU* | *trnT-UGU* | *trnV-GAC*(2) | *trnV-UAC** | *trnW-CCA* |  |
|  |  | *trnY-GUA* | *trnfM-CAU* |  |  |  |  |
| Other genes | Cytochrome c synthesis | *ccsA* |  |  |  |  | 1 |
|  | RNA processing |  |  |  |  |  |  |
|  | Carbon metabolism | *cemA* |  |  |  |  | 1 |
|  | Fatty acid synthesis | *accD** |  |  |  |  | 1 |
|  | proteolysis | *clpP*** |  |  |  |  | 1 |
|  | Other |  |  |  |  |  |  |
| Genes of unknown function | | *ycf1*(2) | *ycf2*(2) | *ycf3*** | *ycf4* |  | 4 |
| Total genes | | | | | | | 108 |

Note:Gene*: Gene with one introns; Gene**: Gene with two introns; Gene(2): Number of copies of multi-copy genes.

Table S8 Chloroplast genome gene information of *I. aquatilis*

| **Classification of genes** | **Subclassification of genes based on functions** | **Gene name** | | | | | **Total numbers** |
| --- | --- | --- | --- | --- | --- | --- | --- |
| Photosynthesis related genes | PhotosystemⅠ | *psaA* | *psaB* | *psaC* | *psaI* | *psaJ* | 5 |
|  | PhotosystemⅡ | *psbA* | *psbB* | *psbC*(2) | *psbD* | *psbE* | 14 |
|  |  | *psbF* | *psbH* | *psbI* | *psbJ* | *psbk* |  |
|  |  | *psbL* | *psbM* | *psbT* | *psbZ* |  |  |
|  | Cytochrome  b/f compelx | *petA* | *petB** | *petD** | *petG* | *petL* | 6 |
|  |  | *petN* |  |  |  |  |  |
|  | ATP synthase | *atpA* | *atpB* | *atpE* | *atpF*（2）* | *atpH* | 6 |
|  |  | *atpI* |  |  |  |  |  |
|  | NADPH dehydrogenase | *ndhA*（2）* | *ndhB*（2）* | *ndhC* | *ndhD* | *ndhE* | 11 |
|  |  | *ndhF* | *ndhG* | *ndhH* | *ndhI* | *ndhJ* |  |
|  |  | *ndhK* |  |  |  |  |  |
|  | Rubisco | *rbcL* |  |  |  |  | 1 |
| Self-replication | Transcription | *rpoA* | *rpoB* | *rpoC1**(2) | *rpoC2* |  | 4 |
|  | Small subunit  of ribosome | *rps11* | *rps12**（2）* | *rps14* | *rps15* | *rps16** | 12 |
|  |  | *rps18* | *rps19（2）* | *rps2* | *rps3* | *rps4* |  |
|  |  | *rps7（2）* | *rps8* |  |  |  |  |
|  | Large subunit  of ribosome | *rpl2*（4）* | *rpl14* | *rpl16** | *rpl20* | *rpl22* | 9 |
|  |  | *rpl23（2）* | *rpl33* | *rpl36* | *rpl32* |  |  |
|  | Translational initiation factor | *infA* |  |  |  |  | 1 |
|  | Ribosomal RNA | *rrn4.5（2）* | *rrn5（2）* | *rrn16（4）* | *rrn23（4）* |  | 4 |
|  | Transfer RNA | *trnA-UGC**(2) | *trnC-GCA* | *trnD-GUC* | *trnE-UUC* | *trnF-GAA* | 30 |
|  |  | *trnG-GCC* | *trnG-UCC** | *trnH-GUG* | *trnI-CAU*(2) | *trnI-GAU**(2) |  |
|  |  | *trnK-UUU** | *trnL-CAA*(2) | *trnL-UAA** | *trnL-UAG* | *trnM-CAU* |  |
|  |  | *trnN-GUU*(2) | *trnP-UGG* | *trnQ-UUG* | *trnR-ACG*(2) | *trnR-UCU* |  |
|  |  | *trnS-GCU* | *trnS-GGA* | *trnS-UGA* | *trnT-GGU* | *trnT-UGU* |  |
|  |  | *trnV-GAC*(2) | *trnV-UAC** | *trnW-CCA* | *trnY-GUA* | *trnfM-CAU* |  |
| Other genes | Cytochrome c synthesis | *ccsA* |  |  |  |  | 1 |
|  | RNA processing | *matK* |  |  |  |  | 1 |
|  | Carbon metabolism | *cemA* |  |  |  |  | 1 |
|  | Fatty acid synthesis | *accD** |  |  |  |  | 1 |
|  | proteolysis | *clpP1*** |  |  |  |  | 1 |
|  | Other | *pafI***(2) | *pafII* | *pbf1* |  |  | 3 |
| Genes of unknown function | | *ycf1*(2) | *ycf2*(2) | *Ycf15*(2) |  |  | 3 |
| Total genes | | | | | | | 114 |

Note: Gene*: Gene with one introns; Gene**: Gene with two introns; Gene(2): Number of copies of multi-copy genes.

Table S9 Chloroplast genome gene information of *I. racemosa*

| **Classification of genes** | **Subclassification of genes based on functions** | **Gene name** | | | | | **Total numbers** |
| --- | --- | --- | --- | --- | --- | --- | --- |
| Photosynthesis related genes | PhotosystemⅠ | *psaA* | *psaB* | *psaC* | *psaI* | *psaJ* | 5 |
|  | PhotosystemⅡ | *psbA* | *psbB* | *psbC*(2) | *psbD* | *psbE* | 14 |
|  |  | *psbF* | *psbH* | *psbI* | *psbJ* | *psbk* |  |
|  |  | *psbL* | *psbM* | *psbT* | *psbZ* |  |  |
|  | Cytochrome  b/f compelx | *petA* | *petB** | *petD** | *petG* | *petL* | 6 |
|  |  | *petN* |  |  |  |  |  |
|  | ATP synthase | *atpA* | *atpB* | *atpE* | *atpF*（2）* | *atpH* | 6 |
|  |  | *atpI* |  |  |  |  |  |
|  | NADPH dehydrogenase | *ndhA*（2）* | *ndhB*（2）* | *ndhC* | *ndhD* | *ndhE* | 11 |
|  |  | *ndhF* | *ndhG* | *ndhH* | *ndhI* | *ndhJ* |  |
|  |  | *ndhK* |  |  |  |  |  |
|  | Rubisco | *rbcL* |  |  |  |  | 1 |
| Self-replication | Transcription | *rpoA* | *rpoB* | *rpoC1**(2) | *rpoC2* |  | 4 |
|  | Small subunit  of ribosome | *rps11* | *rps12**（2）* | *rps14* | *rps15* | *rps16** | 12 |
|  |  | *rps18* | *rps19（2）* | *rps2* | *rps3* | *rps4* |  |
|  |  | *rps7（2）* | *rps8* |  |  |  |  |
|  | Large subunit  of ribosome | *rpl2*（4）* | *rpl14* | *rpl16** | *rpl20* | *rpl22* | 9 |
|  |  | *rpl23（2）* | *rpl33* | *rpl36* | *rpl32* |  |  |
|  | Translational initiation factor | *infA* |  |  |  |  | 1 |
|  | Ribosomal RNA | *rrn4.5S（2）* | *rrn5S（2）* | *rrn16S（4）* | *rrn23S（4）* |  | 4 |
|  | Transfer RNA | *trnA-UGC**(2) | *trnC-GCA* | *trnD-GUC* | *trnE-UUC* | *trnF-GAA* | 27 |
|  |  | *trnG-GCC* | *trnH-GUG* | *trnK-UUU** | *trnL-CAA*(2) | *trnL-UAA** |  |
|  |  | *trnL-UAG* | *trnN-GUU*(2) | *trnP-UGG* | *trnQ-UUG* | *trnR-ACG*(2) |  |
|  |  | *trnS-CGA** | *trnS-GCU* | *trnS-GGA* | *trnS-UGA* | *trnT-UGU* |  |
|  |  | *trnV-GAC*(2) | *trnV-UAC** | *trnW-CCA* | *trnK-UUU** | *trnL-CAA*(2) |  |
|  |  | *trnL-UAA** | *trnL-UAG* |  |  |  |  |
| Other genes | Cytochrome c synthesis | *ccsA* |  |  |  |  | 1 |
|  | RNA processing | *matK* |  |  |  |  | 1 |
|  | Carbon metabolism | *cemA* |  |  |  |  | 1 |
|  | Fatty acid synthesis | *accD** |  |  |  |  | 1 |
|  | proteolysis | *clpP*** |  |  |  |  | 1 |
|  | Other |  |  |  |  |  |  |
| Genes of unknown function | | *ycf1*(2) | *ycf2*(2) | *ycf15*(2) | *ycf3*** | *ycf4* | 5 |
| Total genes | | | | | | | 110 |

Note: Gene*: Gene with one introns; Gene**: Gene with two introns; Gene(2): Number of copies of multi-copy genes.

Table S10. Genetic information of 9 *Impatiens* species

| **species** | **Total Genes** | **Total CDS** | **Total tRNA** | **Total rRNA** |
| --- | --- | --- | --- | --- |
| *I. lateristachys* | 113 | 79 | 30 | 4 |
| *I.siculifer* var. *porphyrea* | 114 | 80 | 30 | 4 |
| *I. apalophylla* | 115 | 81 | 30 | 4 |
| *I. pritzelii* | 113 | 79 | 30 | 4 |
| *I.menghuochengensis* | 113 | 79 | 30 | 4 |
| *I.membranifolia* | 113 | 79 | 30 | 4 |
| *I. qingchengshanica* | 108 | 77 | 27 | 4 |
| *I. aquatilis* | 114 | 81 | 30 | 4 |
| *I.* *racemosa* | 110 | 79 | 27 | 4 |

Table.S11 SSR types and number statistics of chloroplast genomes of *Impatiens*

| **Species** | **Mononucleotide** | **Dinucleotide** | **Trinucleotide** | **Tetranucleotide** | **Pent nucleotide** | **Hexanucleotide** | **Total** |  |
| --- | --- | --- | --- | --- | --- | --- | --- | --- |
| *I. lateristachys* | 61 | 3 | 5 | 5 | 0 | 0 | 74 |  |
| *I.siculifer* var.*porphyrea* | 69 | 3 | 3 | 6 | 0 | 0 | 81 |  |
| *I. apalophylla* | 68 | 5 | 2 | 3 | 0 | 0 | 78 |  |
| *I. pritzelii* | 64 | 5 | 4 | 5 | 0 | 1 | 79 |  |
| *I. chlorosepala* | 82 | 6 | 5 | 9 | 0 | 0 | 102 |  |
| *I.menghuochengensis* | 80 | 5 | 4 | 4 | 0 | 0 | 93 |  |
| *I.membranifolia* | 62 | 7 | 4 | 6 | 0 | 0 | 79 |  |
| *I. qingchengshanica* | | 73 | 4 | 2 | 3 | 0 | 0 | 82 |
| *I. aquatilis* | | 77 | 3 | 2 | 3 | 0 | 0 | 85 |
| *I. racemosa* | | 73 | 3 | 1 | 4 | 0 | 0 | 81 |

*
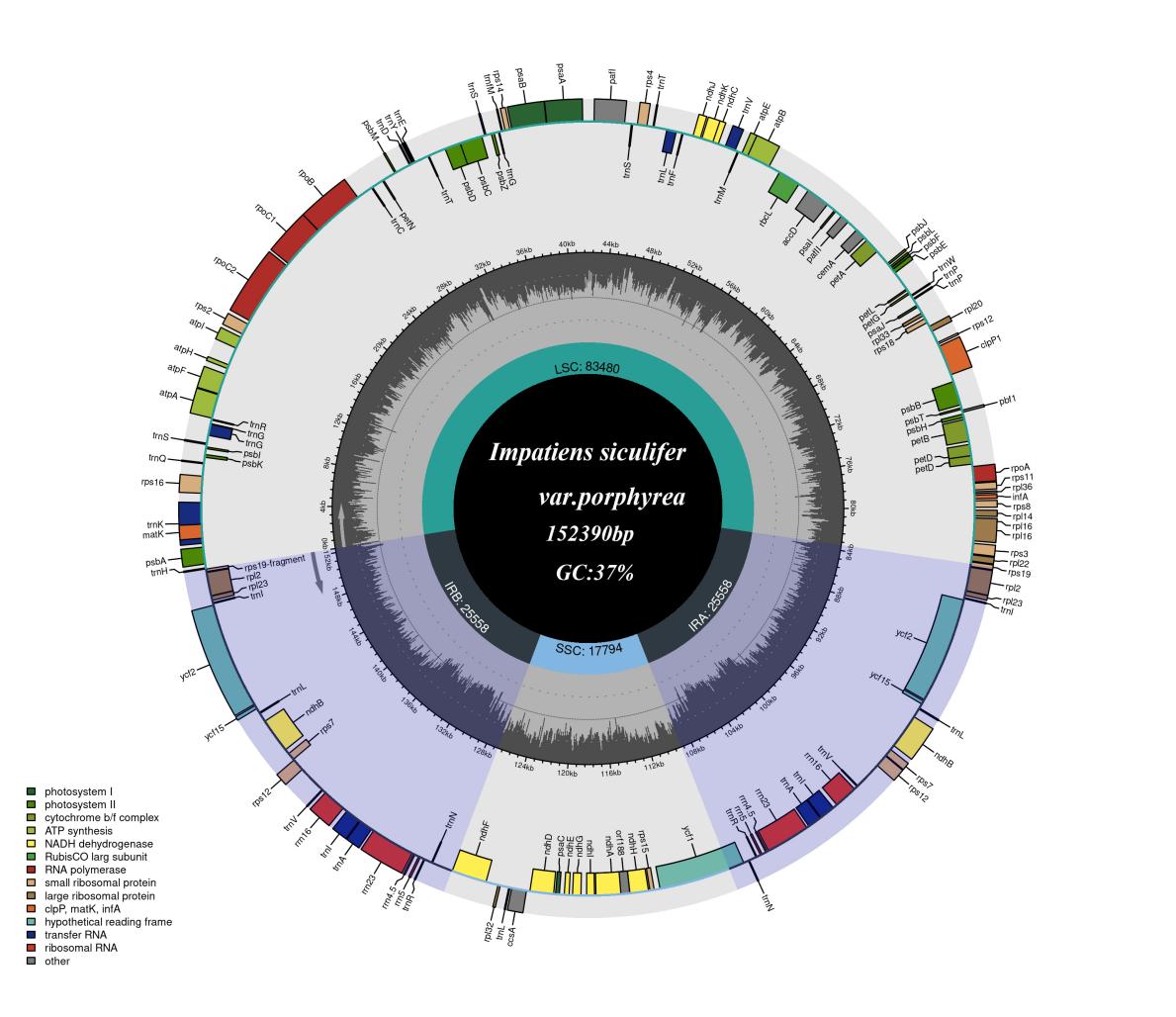

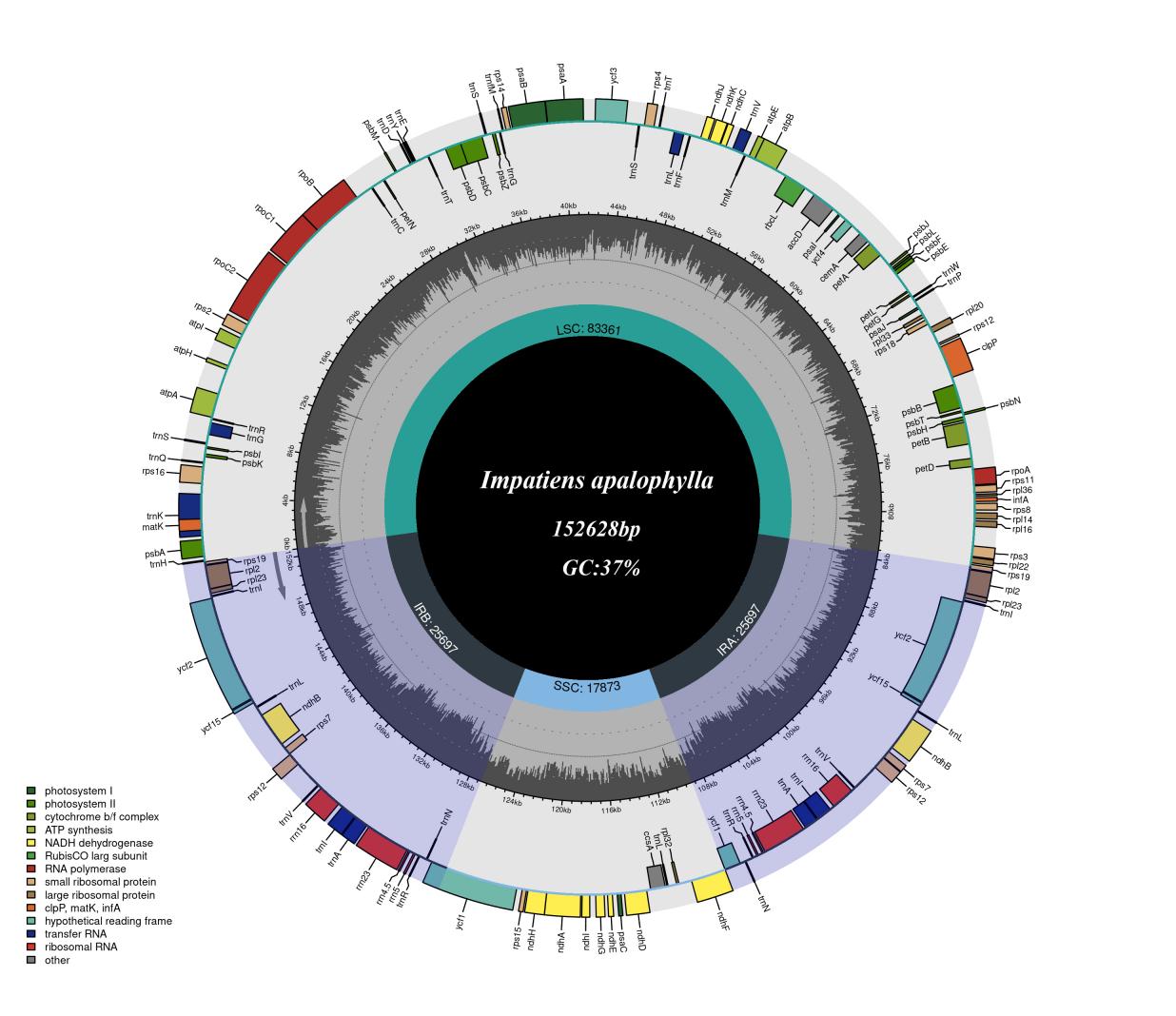
*


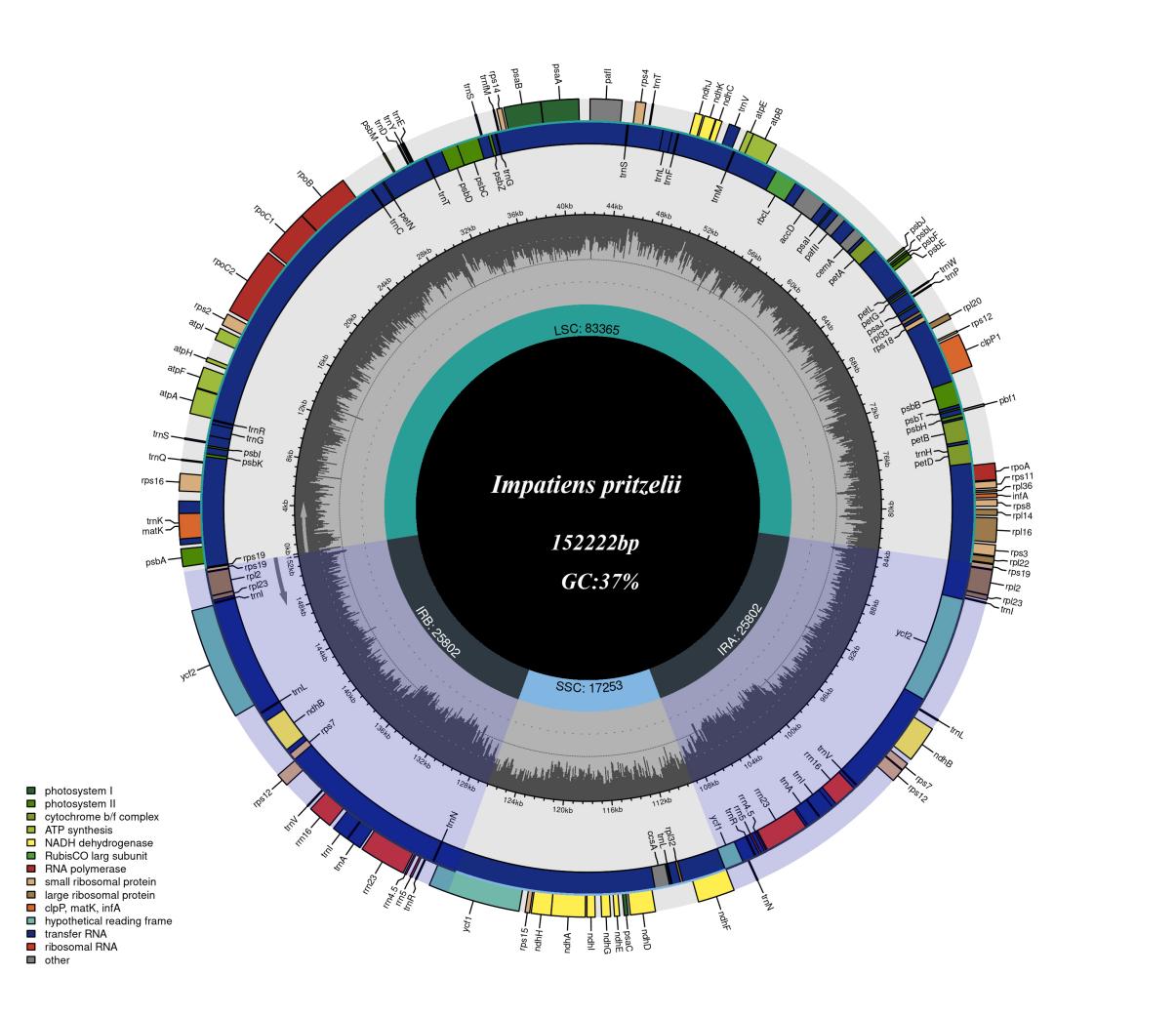


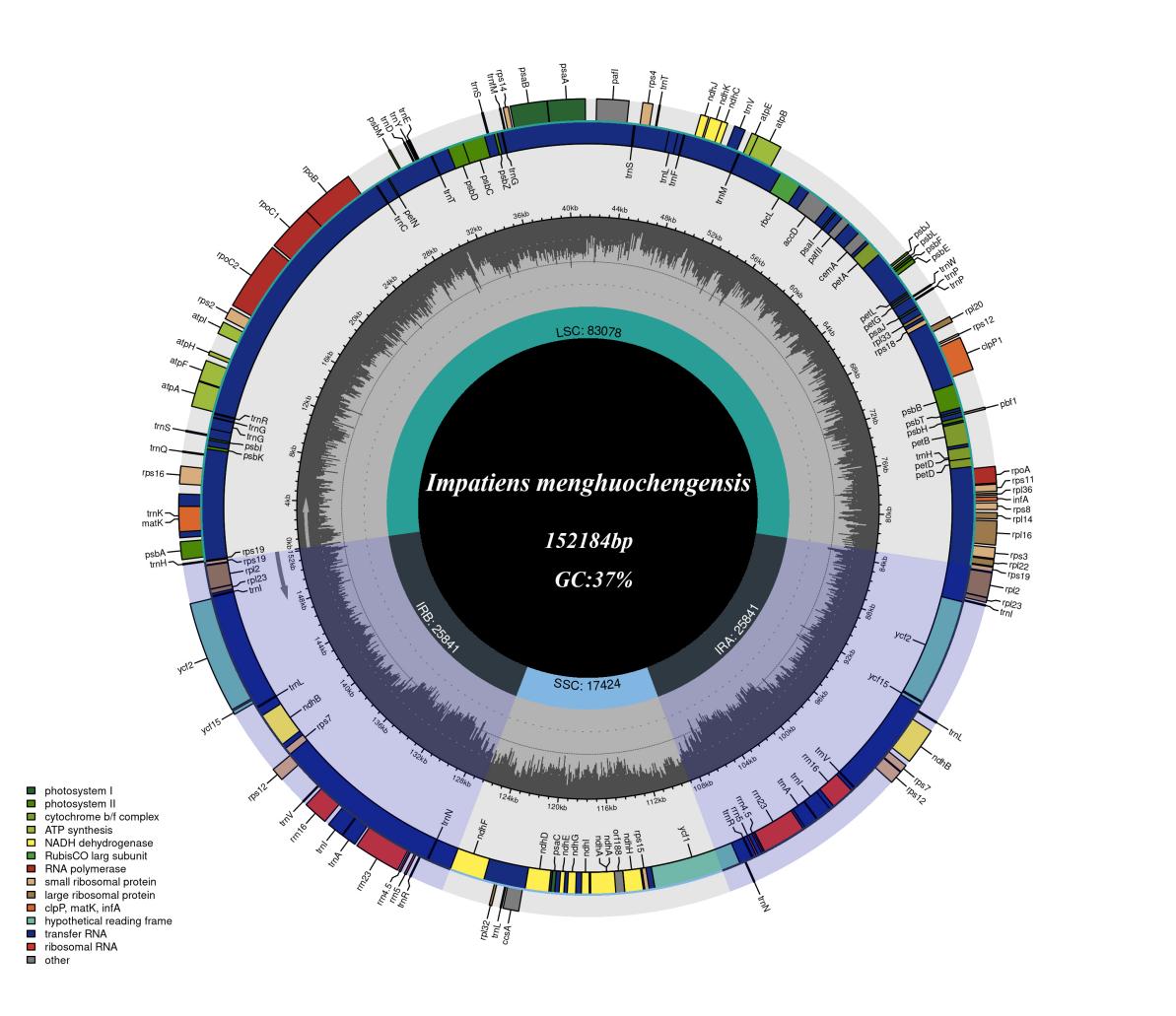

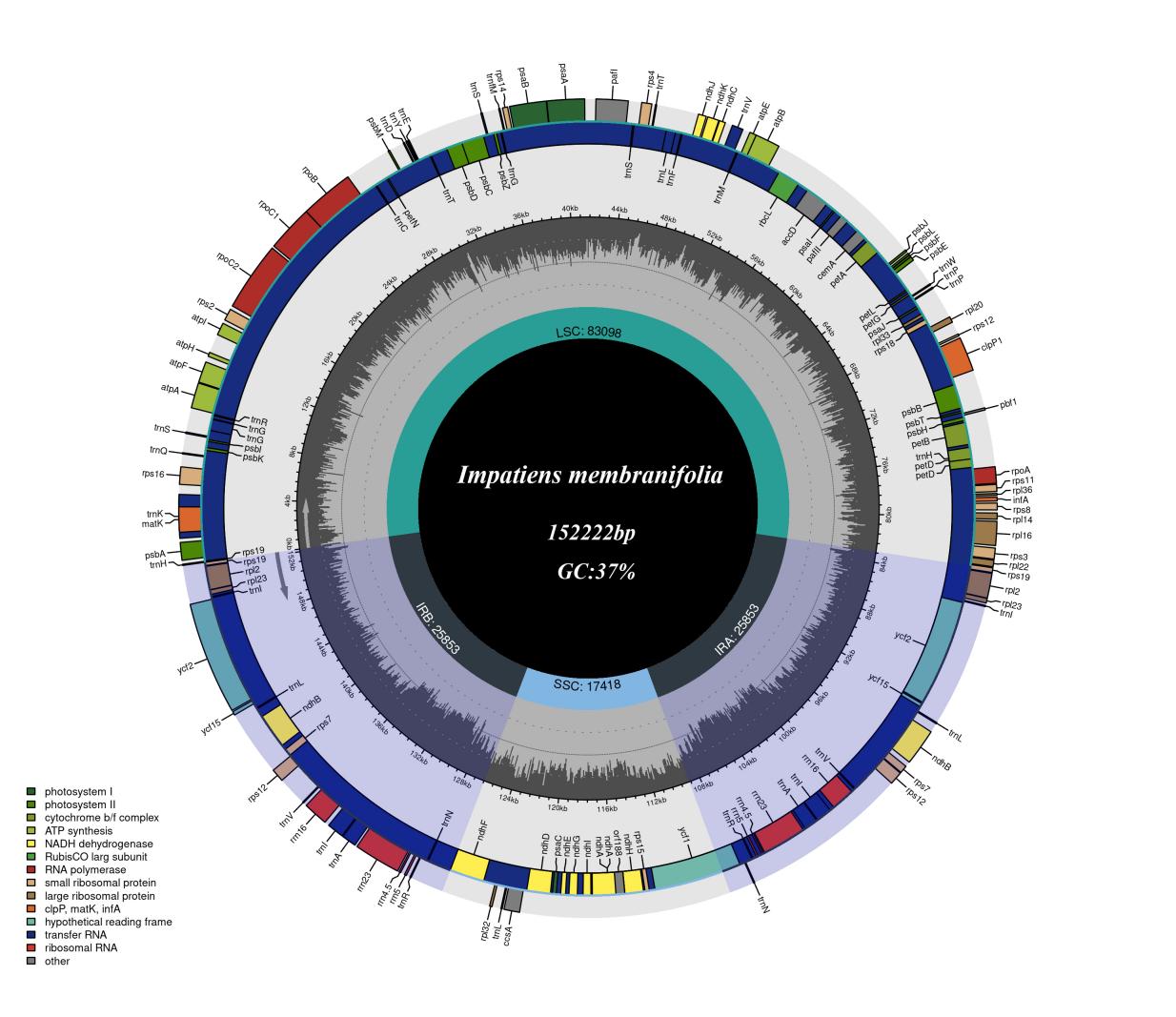


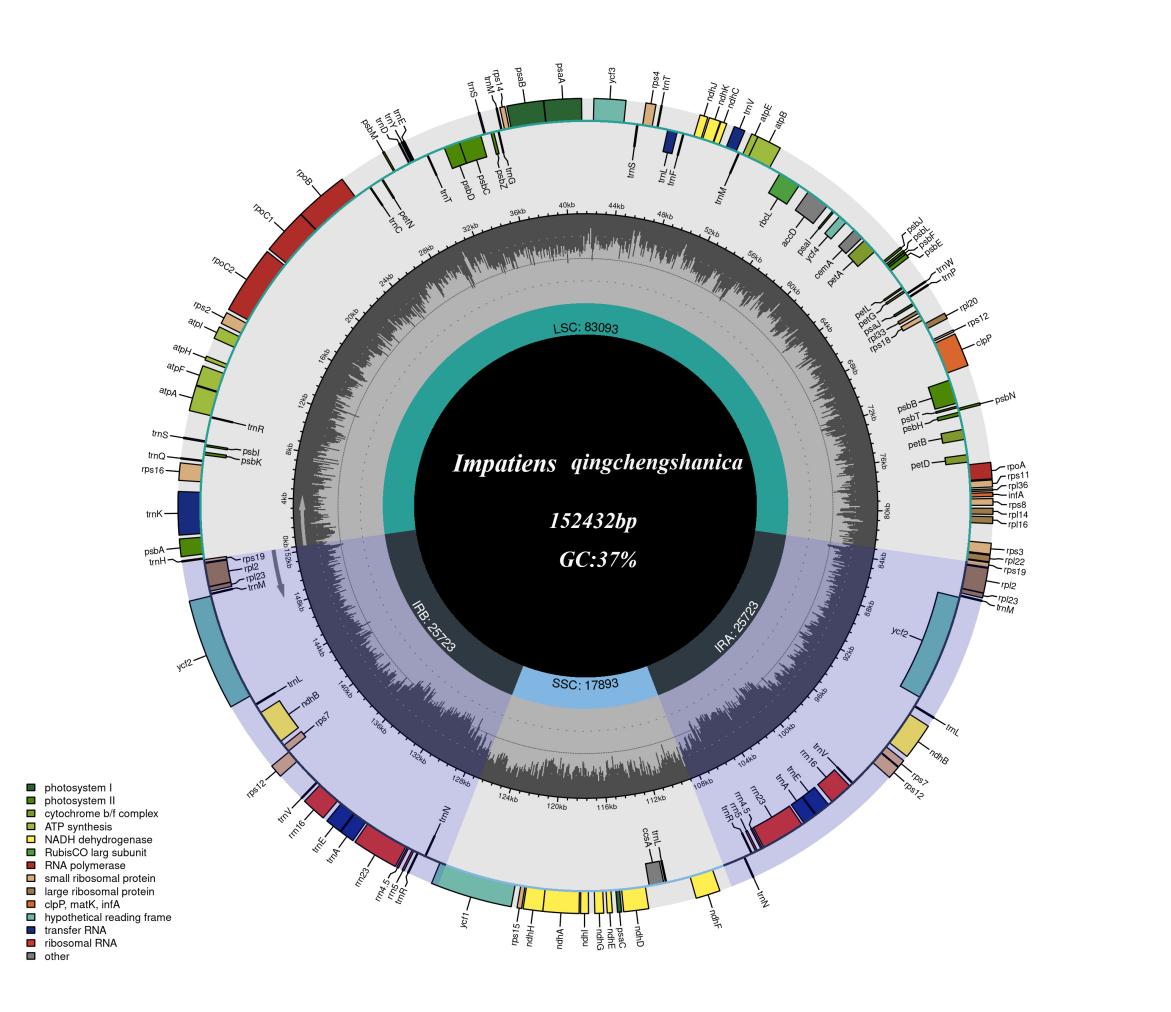

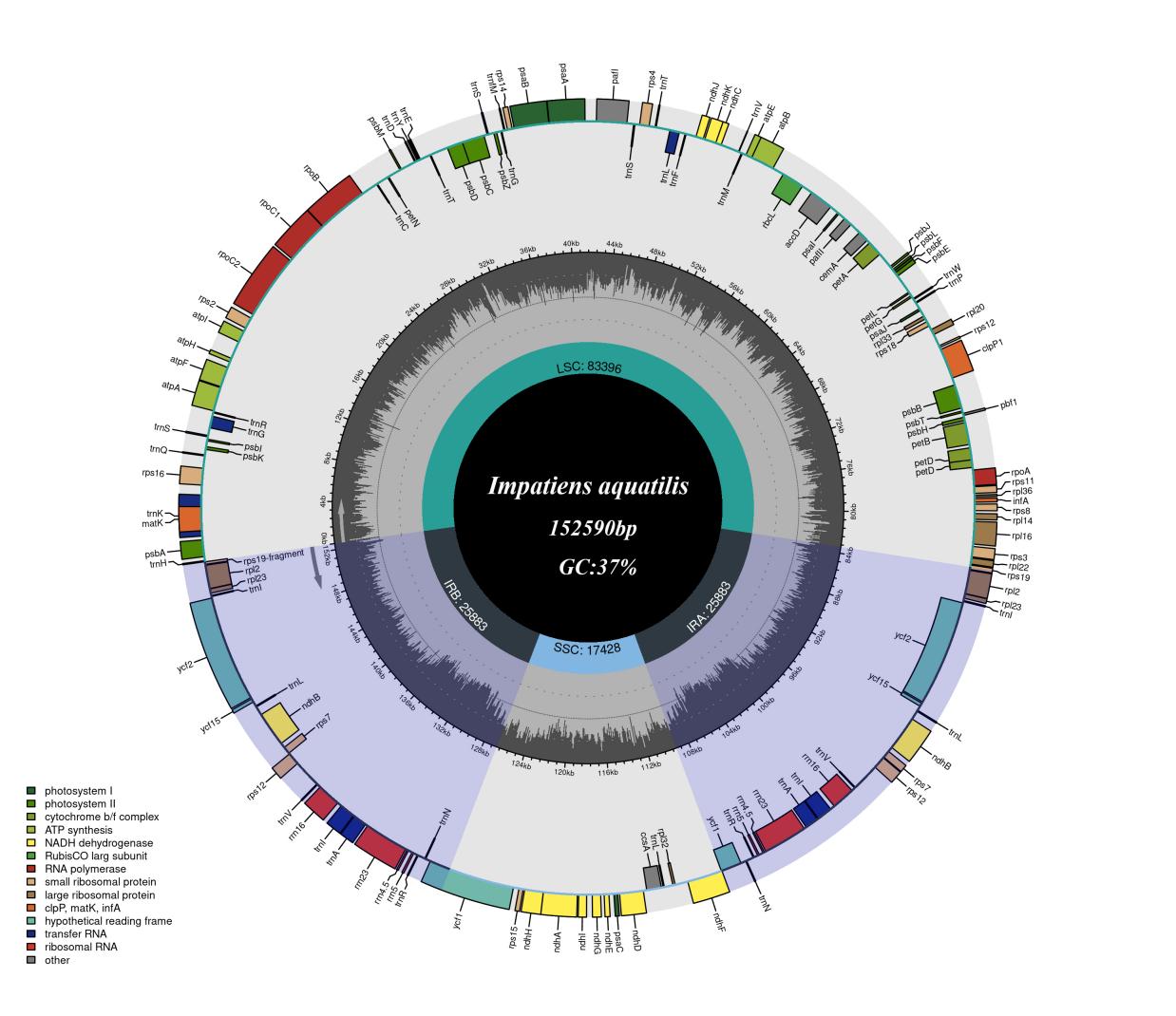

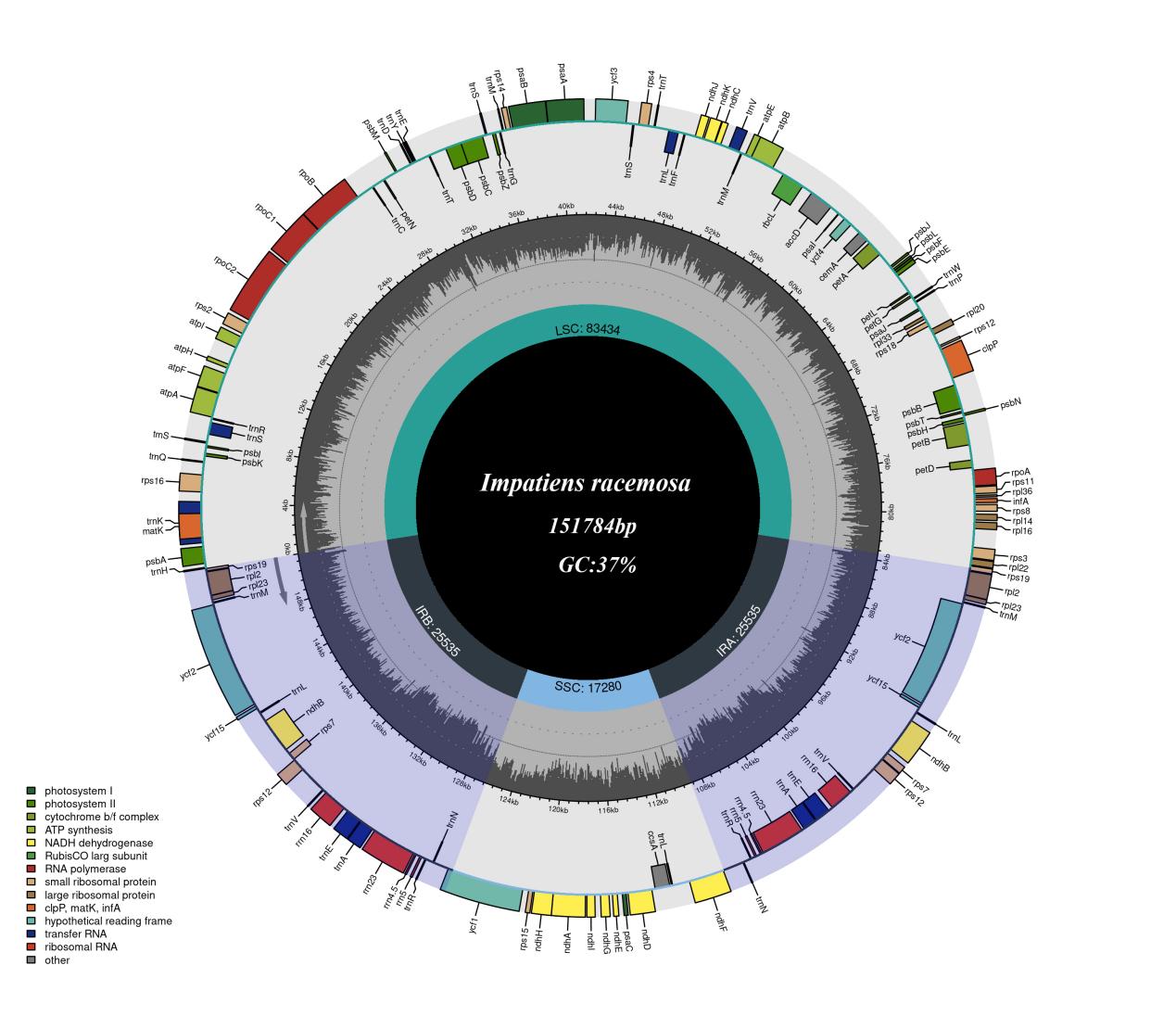


Figure S1 Gene map of *Impatiens* plastomes. The genes that are drawn outside of the map are trancribed clockwise, while those inside are counterclockwise.

Table.S12 The NCBI accession numbers for the species used in constructing the phylogenetic tree are as follows:

| **Species** | **Accession** |
| --- | --- |
| *I. macrovexilla* | NC_060668.1 |
| *I. lasiophyton* | PQ156319.1 |
| *I. piufanensis* | NC_037401.1 |
| *I. davidii* | NC_058801.1 |
| *I. monticola* | NC_058205.1 |
| *I. chlorosepala* | NC_059943.1 |
| *I. mengtszeana* | NC_058215.1 |
| *I. balsamina* | MZ902354.1 |
| *I. uliginosa* | NC_059760.1 |
| *I. cyanantha* | MW464332.1 |
| *I. omeiana* | NC_072171.1 |
| *I. guizhouensis* | NC_059945.1 |
| *Hydrocera triflora* | NC_037400.1 |
| *Primula kwangtungensis* | KX774737.1 |
| *Actinidia chinensis* | KP297245.1 |
